# Landscape-Scale Biodiversity Impacts Analysis of Côte d’Ivoire’s Cocoa Cultivation along Export Supply Chains

**DOI:** 10.1021/acs.est.3c07795

**Published:** 2024-05-18

**Authors:** Shuntian Wang, Stephan Pfister

**Affiliations:** † Department of Civil, Environmental and Geomatic Engineering, Institute of Environmental Engineering, Ecological Systems Design, Swiss Federal Institute of Technology, ETH Zurich, 8093 Zurich, Switzerland; ‡ Department of Humanities, Social, and Political Sciences, Institute of Science, Technology, and Policy (ISTP), Swiss Federal Institute of Technology, ETH Zurich, 8092 Zurich, Switzerland

**Keywords:** biodiversity impacts, supply chain transparency, earth observations, landscape-scale models, cocoa export, sustainable agriculture

## Abstract

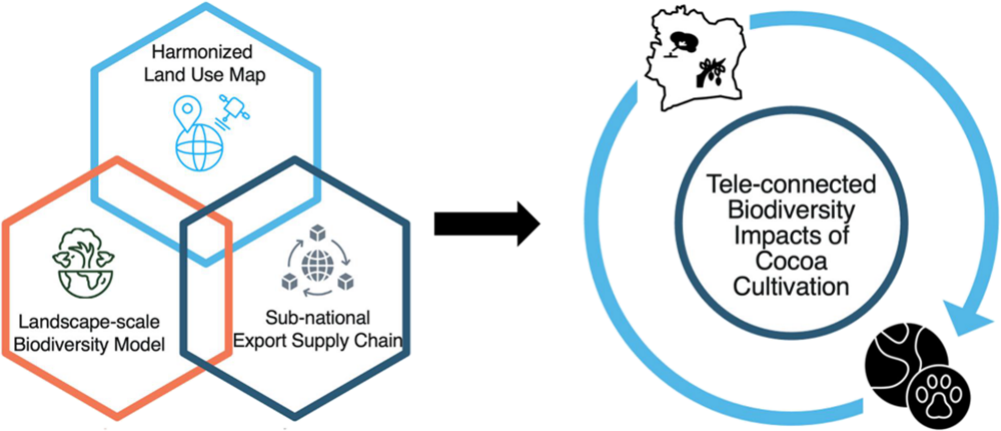

Agricultural land use for export commodities leads to significant biodiversity impacts. A spatially detailed assessment of these impacts is crucial for implementing effective mitigation policies. Using cocoa cultivation and exports in Côte d’Ivoire as an example, we present a novel framework that combines earth observations, enhanced landscape-scale biodiversity models, and subnational export supply chain data sets to track the tele-connected potential biodiversity impacts of export groups and importing countries. We found that cocoa cultivation accounts for ∼44% of the biodiversity impacts in Côte d’Ivoire’s cocoa cultivation areas, with >90% attributable to cocoa exports. The top 10 importing countries account for ∼84% of these impacts. Our method offers improved spatial detail compared to the existing approaches, facilitating the identification of biodiversity impact hotspots. Additionally, the biodiversity impacts of agroforestry cocoa are not always lower compared to full-sun cocoa, especially when agroforestry systems are established in regions of high biodiversity importance. Our transferable framework provides a comprehensive understanding of biodiversity footprint and promotes informed decision-making for sustainable agricultural production, processing, and trade. Our framework’s application is currently constrained by the scarcity of detailed supply chain data sets; we underscore the urgent need for improved supply chain transparency to fully unlock the framework’s potential.

## Abstract 2

Most research on the land use-related biodiversity impacts assessment along agricultural export supply chains is limited in terms of spatial detail. We take cocoa cultivation in Côte d’Ivoire as an example, illustrating a novel method to track the biodiversity footprint at the landscape-scale precisely along the export supply chain.

## 1 Introduction

The global food system is the leading driver of biodiversity loss, as agricultural production threatens ∼86% of the world’s species.^1^ This biodiversity loss is primarily caused by converting natural terrestrial habitats to agricultural land.^2−6^ Additionally, driven by the complex global supply chains, about 1/3 of the biodiversity loss of the agri-food sector is embodied in commodities trade, exacerbating inequities in shared environmental responsibility.^6−8^ Therefore, assessing the biodiversity impacts of agricultural land use along supply chains is critical for further impact mitigation. Three steps are essential for such an assessment: (1) accurately mapping the land use status of production areas; (2) modeling land use-related biodiversity impacts in production areas; and (3) quantifying tele-connected impacts driven by demand.^9^

### 1.1 Challenges in Land Use Mapping

Many studies have combined earth observations, land use statistics, and scenario analysis to obtain land use maps at regional or global scales.^10−17^ These maps are crucial in land use-related biodiversity impact assessment (BIA).^3,4,18−21^ However, commonly used land use maps, such as HYDE 3.2^16^ and LUH2,^15^ rely on limited observations and represent land use types as percentages within coarse grid cells. By allocating land use directly to finer grid cells instead of integrating it as a percentage, the HILDA+^13^ and GLOBIO4^12^ maps provide global land use distributions at ∼1 km and ∼300 m spatial resolution, respectively. These enhanced maps facilitate spatially enhanced BIA.^12,22^ For BIAs focused on specific crop cultivation, dedicated crop maps are required. These maps are typically produced by predicting crop suitability and spatial distribution by combining climate, soil, and topography data sets, albeit at a coarser spatial resolution.^10,23,24^ With advancements in earth observations, high-resolution (∼10 m) land cover and crop maps are available,^25−28^ providing the possibility of a landscape-scale BIA. However, there are no generally accepted guidelines for converting high-resolution land cover maps into land use maps and remotely sensed crop maps have not yet been integrated into BIA.^29,30^

### 1.2 Metrics for Quantifying Land Use-Related Biodiversity Impacts

Different metrics have been used for BIA. The potentially disappeared fraction (PDF) of species is a commonly used metric in life cycle assessment (LCA) to measure land use-related biodiversity impacts.^3,4,31−33^ It quantifies the potential extinction of species due to land occupation and transformation. Chaudhary et al.^3^ modeled PDF characterization factors (CFs) from land use for four taxa groups across 804 terrestrial ecoregions. These CFs are recommended by the United Nations Environment Programme (UNEP) life cycle initiative. However, PDF is typically retrieved based on the homogeneity assumption for the same land use type within a specific ecoregion, which limits the spatial detail of BIA. The biodiversity intactness index (BII) measures the proportion of native species and their abundances that remain at a grid cell level, reflecting how native species populations persist despite human pressures.^34−36^ Landscape factors such as habitat fragmentation and connectivity are not explicitly considered in BII. The mean species abundance (MSA), which is used in the GLOBIO model that measures biodiversity like BII, assesses how human activities affect species abundance compared to their original state. Compared to BII, MSA takes into account landscape factors such as road disturbance and habitat fragmentation.^12,18,37^ The MSA and BII serve as local indicators tailored to assess the biodiversity intactness at specific sites. Their utility for broad-scale comparisons or cumulative assessments is limited if regional differences are not considered. Biodiversity impact metric (BIM)^38^ integrated biodiversity intactness (MSA) with the biodiversity importance indicator, which allows for direct comparisons of impacts at different locations. Here, we choose BIM for the landscape-scale BIA, as it combines importance weighting of the PDF approach with landscape details from MSA.

### 1.3 Supply Chain Data Sets in Tracking Tele-connected Biodiversity Impacts

Detailed supply chain data sets are essential to track the tele-connected biodiversity impacts. Environmentally extended multiregional input–output tables (EE-MRIO) are widely used as a tool to map the biodiversity impacts along supply chains.^5,6,8,19,39−41^ However, available EE-MRIO usually covers agri-food supply chains as several aggregated sectors. Trase provides detailed export supply chain information for specific commodities in a limited number of regions, including cocoa export from Côte d’Ivoire, allowing for detailed tracking of the tele-connected impacts of export groups and importing countries.^42^ To couple the landscape-scale biodiversity impacts of cocoa cultivation quantified in this study, we selected the Trase data set to track biodiversity impacts along cocoa export supply chains.

### 1.4 Research Questions and Case Selection

In this study, we aimed to investigate the following three questions to improve BIA along supply chains:

1. How can earth observations be harmonized into land use maps using appropriate supplementary and reasonable allocation rules (Section 3.1)?
2. How can biodiversity impacts be modeled at the landscape scale and the land use-related biodiversity impacts for specific crops be quantified (Section 3.2)?
3. How can biodiversity impacts be linked to the export supply chain and the tele-connected biodiversity impacts be tracked (Section 3.3)?

We selected cocoa exports from Côte d’Ivoire as a case study for two key reasons. First, Côte d’Ivoire is the world’s leading cocoa-producing country and the largest exporter. Cocoa accounts for 40% of the country’s exports and 15% of its GDP.^28,42−44^ This substantial production has led to significant deforestation and biodiversity loss. For example, Côte d’Ivoire encompasses a large part of the Upper Guinean Forest, a global biodiversity hotspot currently under severe threat from cocoa expansion.^45^ Against this background, it is urgent to investigate the biodiversity impacts along the cocoa export supply chain. Second, the availability of the remotely sensed cocoa map and detailed export supply chains provided by Trase enables the application of our research framework to effectively assess these pressing biodiversity challenges.

## 2 Methods

**Figure 1:**
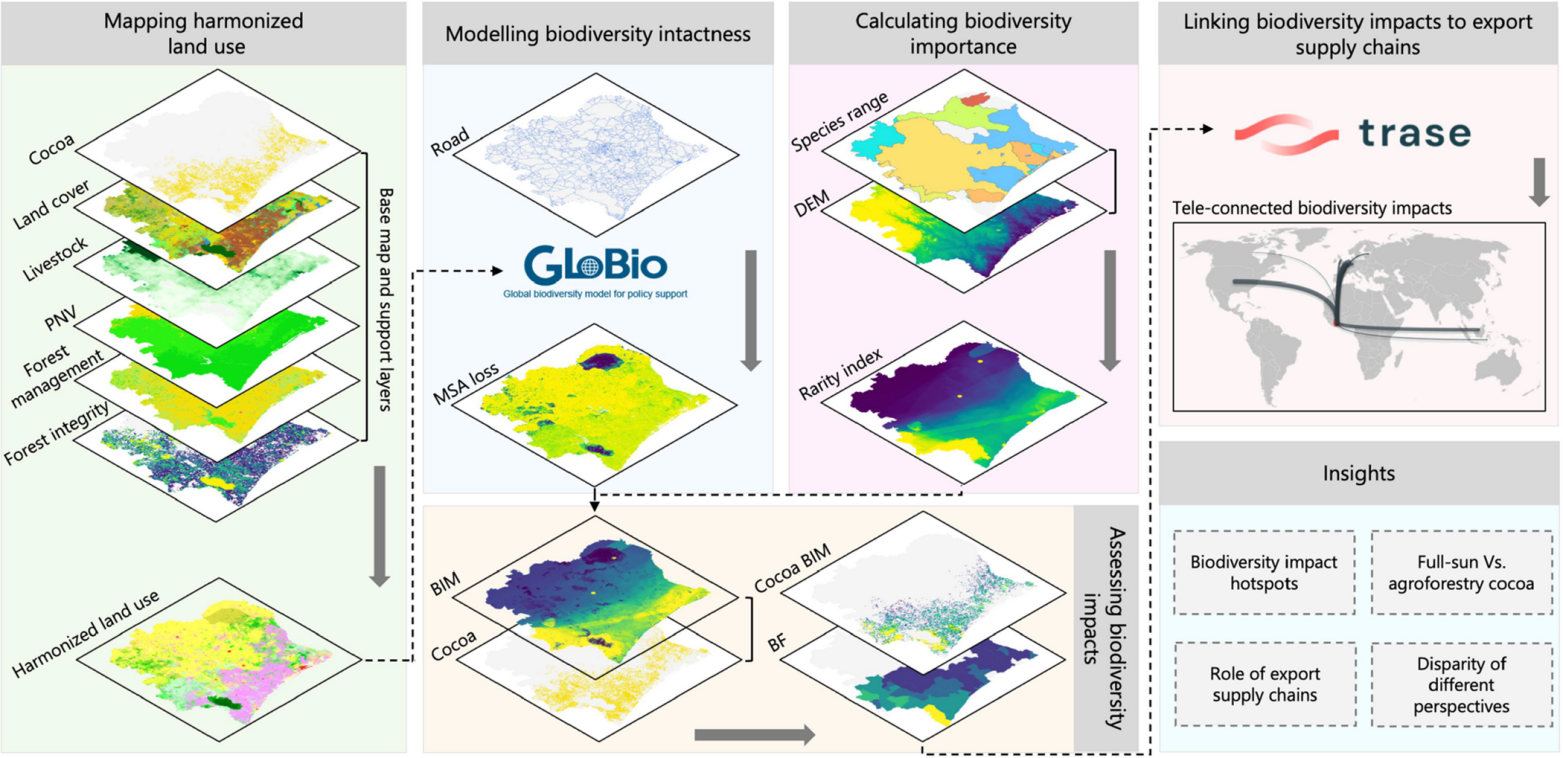
Methodological framework of the landscape-scale biodiversity impacts analysis of cocoa cultivation in Côte d’Ivoire along export supply chains: (1) the process begins with the construction of a harmonized land use map for Côte d’Ivoire based on up-to-date earth observations and land use statistics; (2) the harmonized map is then fed into the GLOBIO-InVEST model to estimate the loss of biodiversity intactness caused by cocoa cultivation, using mean species abundance (MSA) as an indicator; (3) biodiversity importance is calculated at the grid cell scale using species range and digital elevation model (DEM) data; (4) biodiversity impact metric (BIM) is then calculated by multiplying the biodiversity intactness loss and biodiversity importance. (5) Trase supply chain data allows for the assessment of tele-connected biodiversity impacts. We highlight findings on the identification of biodiversity impact hotspots, the role of export supply chains in driving biodiversity impacts, the comparison of biodiversity impacts between full-sun and agroforestry cocoa cultivation, and the disparity of biodiversity impacts from different perspectives.

We first developed a harmonized land use map for Côte d’Ivoire, followed by an assessment of the biodiversity impacts of cocoa cultivation using landscape-scale biodiversity models. We then linked the biodiversity impacts to subnational export supply chains to track the tele-connected impacts of export groups and importing countries. The methodological framework of this study is shown in Figure 1.

### 2.1 Developing a Harmonized Land Use Map for Côte d’Ivoire

Before modeling the biodiversity impacts of specific human land use activities (e.g., cocoa cultivation) at the landscape scale, we first need to obtain a detailed land use map of Côte d’Ivoire. Based on up-to-date remotely sensed data sets, land use statistics, and land use allocation rules, we developed a harmonized land use map for Côte d’Ivoire, encompassing nine land use types (land use categories and definitions are shown in Table S1) at ∼10 m spatial resolution. This map comprehensively represents land use status with enhanced detail and serves as the basic input for biodiversity intactness modeling.

The land use allocation process requires three key inputs: boundary conditions, support layers, and a land cover base map. Boundary conditions are used to limit the total area of certain land use types or prohibit certain land use types from occupying certain areas. Support layers provide supplementary data that offers valuable information to guide the distribution of specific land use types. The base map provides information on where and what types of land use can be allocated. The BNETD 2016 land cover map provided by the National Bureau of Technical and Development Studies in Côte d’Ivoire serves as the base map (Figure S4). This map is the most detailed local land cover map available in Côte d’Ivoire, based on satellite remote sensing and field sampling. Details of the key inputs we used in the land use allocation process are listed in Table S2.

The land use allocation process follows predefined priorities of land use types (see Figure S1). We assigned the highest priority to cocoa cultivation. Cocoa grid cells were initially allocated using a remotely sensed cocoa map.^46^ These grid cells were then further allocated to full-sun and agroforestry cocoa by incorporating information from the BNETD 2016 map. Following this, other agricultural grid cells in the BNETD 2016 map were allocated as either “Low-input agriculture” or “Agroforestry”. For biodiversity intactness modeling, we reallocated full-sun and agroforestry cocoa as “Low-input agriculture” and “Agroforestry”, respectively.

For pasture allocation, we adopted the methodology used by GLOBIO4,^12^ setting the total area reported by FAOSTAT^43^ as our boundary condition. The Gridded Livestock of the World database (GLW v4)^47^ was utilized to calculate the pasture suitability values. We then allocated herbaceous and shrub land types from the BNETD 2016 map to pastures. This allocation began with grid cells of the highest suitability and continued until the total designated pasture area was accounted for. Additionally, the potential natural vegetation (PNV)^48^ map is employed to classify pasture types.

The forest grid cells in the BNETD 2016 map were then allocated to different forest types using two support layers: the Global Forest Management Data (GFMD)^49^ to specify the spatial distribution of “Primary forest” and “Plantation forest” and the Forest Landscape Integrity Index (FLII)^50^ to allocate “Lightly used natural forest” and “Secondary forest”. Finally, the other unallocated vegetation grid cells are allocated to “Primary vegetation”. The bare land and infrastructure grid cells in BNETD 2016 map are allocated to “Built-up areas”.

Due to data availability limitations, the support layers and base map used in the land use allocation process span from 2015 to 2020. To reduce spatial inconsistency, all support layers are resampled to the spatial extent of the base map. The detailed land use allocation method is described in Methods S1. The flowchart for land use allocation is shown in Figure S1.

### 2.2 Modeling Land Use-Related Biodiversity Impacts of Cocoa Cultivation

#### 2.2.1 Modeling Biodiversity Intactness

We used GlOBIO-InVEST^51^ to model biodiversity intactness. GLOBIO-InVEST uses MSA to indicate the biodiversity intactness under the pressure of land use changes, habitat fragmentation, and infrastructure.^12,18^ MSA ranges from 0 to 1. A score of 1 indicates that the species composition is completely intact, while a score of 0 indicates that all original species are locally extinct. The MSA calculation integrates the effects of multiple pressures using spatially explicit land use data to assess the impact of human activities across multiple scenarios. Cause-effect parameters for these pressures are derived from extensive meta-analyses and expert perspectives. We imported the harmonized land use map constructed in Section 2.1 and the infrastructure data set from the Global Roads Inventory Project (GRIP)^52^ into GLOBIO-InVEST for modeling MSA. The model’s cause-effect parameters setting and further details can be found in Methods S2.

To calculate the impacts of cocoa cultivation on biodiversity intactness, we employed a widely accepted method within the LCA framework. This approach involves quantifying the impact of a specific land use on biodiversity by comparing the relative biodiversity differences between the land use in question and a natural reference scenario.^3,53−55^ Within this framework, we overlook the temporal dynamics of land use and biodiversity, to assign a fixed “biodiversity score” to a specific biodiversity pressure. We simulated MSA in the presence and absence of cocoa cultivation, where cocoa grid cells were replaced by “Primary forest” in the harmonized land use map to reflect the reference precultivation conditions. The impacts of cocoa cultivation on biodiversity intactness are then defined as MSA loss between the precultivation and the cocoa cultivation scenario.

#### 2.2.2 Calculating Biodiversity Importance

MSA is a local metric that does not incorporate global endemic species richness, an aspect recommended by the UNEP life cycle initiative for assessing land use-related biodiversity impacts.^53,56^ To address this, we introduced the rarity index (RI) to indicate biodiversity importance. We calculated the RI for all terrestrial grid cells in Côte d’Ivoire by following two steps:

(1) Calculating aggregated rarity-weighted richness (RWR): the RWR for each species is defined as the ratio of the range area in a specific grid cell to the species’ global range area. We calculated RWR at ∼100 m spatial resolution for all terrestrial species within the four fully assessed taxa (mammals, reptiles, amphibians, and birds) by the IUCN Red List of Threatened Species,^57^ utilizing the species spatial range maps from the IUCN Red List 2022–2 and Birdlife 2022.^58^ Global multiresolution terrain elevation data 2010 (GMTED2010)^59^ was employed to filter the suitable elevation range of each species. Each grid cell’s aggregated RWR is the sum of the RWR of each species.

(2) Transforming aggregated RWR to RI: The distribution of aggregated RWR is bimodal and exhibits right-skewness (Figure S2c). Following the approach of Tayleur et al.,^38^ we adapted the aggregated RWR to the RI for a more intuitive representation of biodiversity importance and to have a normalized factor that varies around 1 (i.e., the RI can be interpreted as a biodiversity equivalent factor of an average area in Côte d’Ivoire). We first applied a logarithmic transformation to reduce skewness. Then, we divided the scores by the mean, giving a score of 1 to grid cells with average aggregated RWR. Coefficients less than or greater than 1 indicate below-average or above-average biodiversity importance, respectively. Finally, the RI was bound to a minimum value of 0.05 to avoid negative or zero values. The aggregated RWR and RI distributions are shown in Figure S2.

#### 2.2.3 Quantifying the Land Use-Related Biodiversity Impacts of Cocoa Cultivation

To integrate biodiversity intactness (MSA) and importance (RI) indicators, we adapted Tayleur et al.’s BIM definition^38^ to calculate land use-related biodiversity impacts, as depicted by the flowchart in Figure S3. The land use-related biodiversity impacts of all human pressures (BIM_total_) in grid cell *i* was calculated as follows:
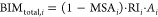
1Here, “1” is the MSA value indicates full biodiversity intactness without human pressures, MSA_*i*_ is the MSA value of grid cell *i* under current land use, RI_*i*_ is the RI of grid cell *i* and *A*_*i*_ is the area [ha] of grid cell *i*.

The land use-related biodiversity impacts of cocoa cultivation (BIM_cocoa_) in grid cell *i* was calculated by multiplying the MSA loss between the precultivation and the cocoa cultivation scenario with RI, and the area of grid cell *i* as follows:
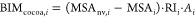
2Here, MSA_nv,*i*_ is the MSA value in cocoa grid cell *i* in the absence of cocoa precultivation (i.e., primary forest).

The BIM is measured in biodiversity-weighted hectares [ha], serving as a proxy for the potential biodiversity loss in Côte d’Ivoire if land use activities (e.g., cocoa cultivation) are maintained. This measure considers the relative importance of biodiversity loss due to land use by assigning a weight to each affected hectare that reflects how much biodiversity has been potentially lost and how important the loss is relative to other areas. Unlike the original BIM definition, which focuses solely on specific land use types, our revised BIM metric incorporates the biodiversity response of landscape factors. For instance, planting cocoa around the forest has higher BIM_cocoa_ compared to agricultural land, despite identical RI and the same cocoa cultivation area. This distinction arises from the more significant biodiversity impacts of cocoa cultivation-induced fragmentation in forests compared to agricultural land.

Before connecting the land use-related biodiversity impacts of cocoa cultivation to export supply chains, it is essential to determine the biodiversity impacts per ton of cocoa produced. Due to the lack of spatially explicit cocoa yield statistics for Côte d’Ivoire, we take Renier et al.^42^’s approach to estimating the cocoa yield by the weight of the relative suitability. We first downscaled the cocoa suitability layer^60^ to the spatial content of the cocoa map and scaled the yields [ton**·**ha^–1^**·**year^–1^] of grid cell *i* (*Y*_*i*_) to match Côte d’Ivoire’s total cocoa production from the 2018 to 2019 season as follows:
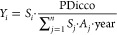
3

In this equation, *S*_*i*_ is the cocoa suitability value of grid cell *i*, PDicco is the total cocoa production [ton] as reported by the International Cocoa Organization (ICCO),^61^*n* is the total number of cocoa grid cells, *j* is each cocoa grid cell, and year is the land occupation time corresponding to the total cocoa production (the value here is 1, representing the 2018–2019 season). The unit of yields is [ton**·**ha^–1^**·**year^–1^], representing the annual cocoa production per ha. We then defined the biodiversity impacts per ton of cocoa produced as biodiversity factor (BF_cocoa_) in grid cell *i* as follows:
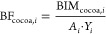
4

Since the supply chain data are only available at a departmental scale, we calculated the BF_cocoa_ [ha**·**year**·**ton^–1^] of each department as the average value of BF_cocoa_. We also computed the BF_cocoa_ for each department’s full-sun and agroforestry cocoa cultivation separately for comparative analysis. For the biodiversity impacts of cocoa production in a given year, the unit of BIM_cocoa_ calculated by multiplying BF_cocoa_ with cocoa production is [ha·year], which adds the time dimension to the original BIM metric. This is commonly adopted in LCA studies,^3,22,62^ indicating that longer periods of land occupation may result in more significant biodiversity impacts.

BIM_cocoa_ is an absolute metric for quantifying the biodiversity impacts of cocoa cultivation, allowing direct comparisons of biodiversity impacts in different locations in Côte d’Ivoire. However, an overall perspective is only one part of a comprehensive understanding of biodiversity impacts. For instance, identifying a department as optimal for cocoa cultivation based on its lower BF_cocoa_ values does not account for the potentially irreversible extinction of local species due to excessive cocoa cultivation. To address this, we introduce the relative BIM_cocoa,%_ metric, which represents the share of BIM_cocoa_ in the department’s total biodiversity impacts (BIM_total_). It provides a more nuanced understanding of the relative contribution of cocoa cultivation to the biodiversity loss in each department and underscores the need for a balanced approach that aligns national biodiversity objectives with local ecological considerations.

### 2.3 Linking Biodiversity Impacts of Cocoa Cultivation to Export Supply Chain

We utilized the subnational Trase data set from the Côte d’Ivoire cocoa SEI-PCS model^42^ to link the biodiversity impacts of cocoa cultivation to export supply chains. The SEI-PCS model estimates trade volumes from disclosed cooperatives and determines trade volumes by multiplying farmers’ number with estimated cocoa production per farmer.^42^ The Trase data set links the 2019 cocoa production to specific exporter groups and importing countries. We calculated the BIM_cocoa_ for exporter groups and importing countries by multiplying the BF_cocoa_ (as detailed in Section 2.2.3) of each department with the volume exported by each trader and imported by each importing country. The traceability of cocoa production is compromised due to two key factors: first, a significant portion of the supply is indirectly sourced through local intermediaries, leaving the origin of the cocoa unknown. Second, approximately a third of the cocoa is exported by nontransparent traders who fail to disclose their suppliers. As a result, 54.4% of cocoa production cannot be traced back to its production departments. For supply chains that cannot be traced back to the production department, we follow the approach of Renier et al.^42^ to estimate biodiversity impacts by multiplying the trade volume with the average BF_cocoa_, weighted by the proportion of total untraced volume in each department. The biodiversity impacts per ton of cocoa imported from Côte d’Ivoire for each importing country (k) is expressed as BF_cocoa,import,k_.

### 2.4 Uncertainties and Comparisons

Our approach acknowledges multiple sources of uncertainty. We evaluated these uncertainties qualitatively and where we could quantitatively. We compared the high-resolution land use map constructed in this study with two widely used land use maps and visually checked robustness with Sentinel-2 cloudless satellite true-color image.^63^ Sensitivity analysis was conducted to assess uncertainties arising from the GLOBIO-InVEST model. This involved determining the lower and upper bounds/relative errors for the biodiversity impacts distribution, using different land use maps (GLOBIO4, HILDA+), using 95% confidence intervals, and varying the simulation parameters via standard deviation. To demonstrate the insights provided by our novel approach, we compared our findings with results obtained from the UNEP life cycle initiative^3,56^ recommended CFs for land use-related biodiversity impact assessment (focusing on global species loss, using global PDF as the biodiversity indicator). The specific steps for comparative analysis are detailed in Methods S3. The results of the uncertainty analysis are in Results S3.

## 3 Results

### 3.1 Harmonizing Local and Global Earth Observations for BIA

**Figure 2:**
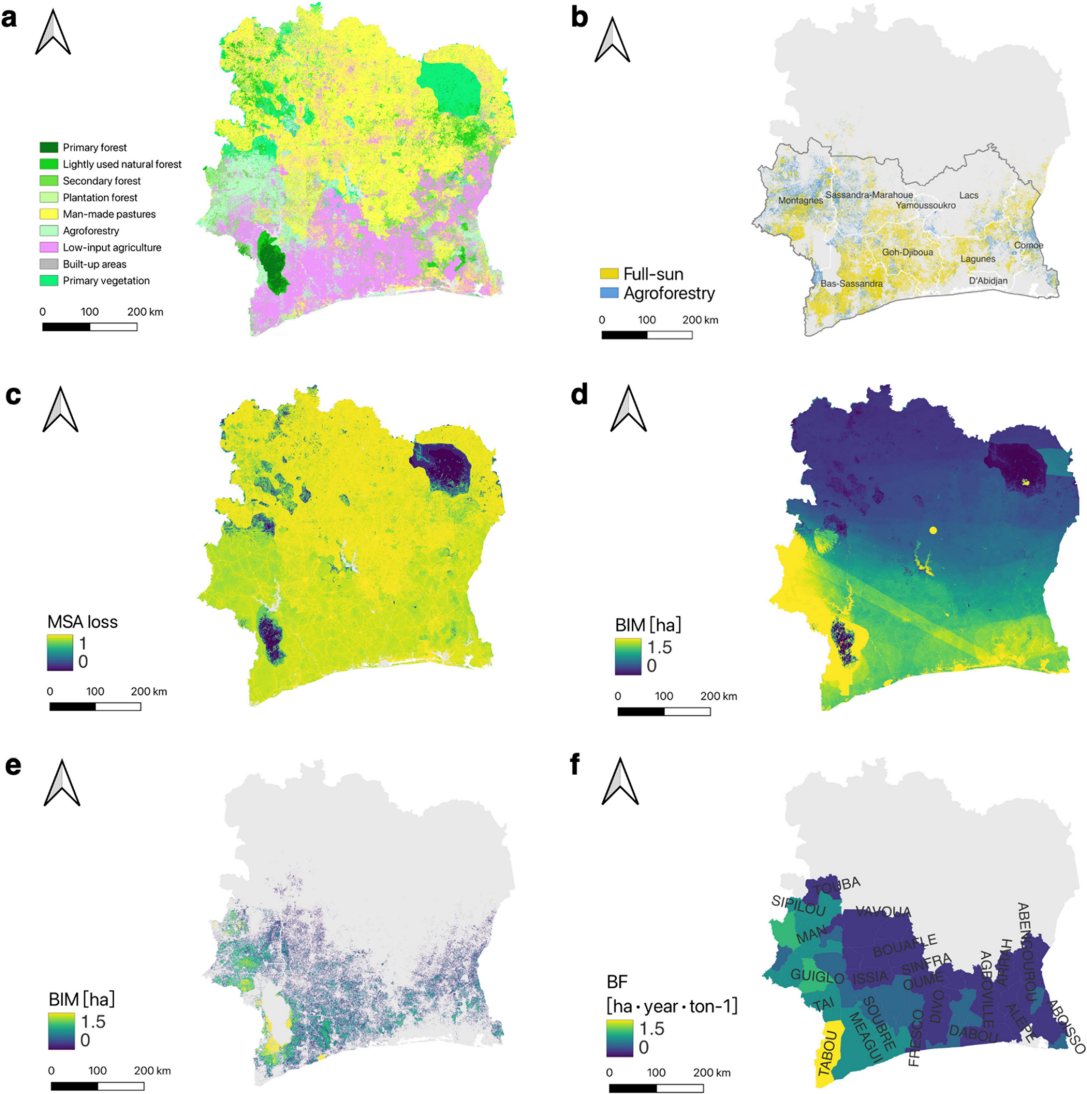
(a) Harmonized land use map of Côte d’Ivoire developed in this study. (b) Spatial distribution of cocoa cultivation areas and types; the gray border is the cocoa climatically suitable growing regions. (c) Spatial distribution of MSA loss in Côte d’Ivoire. (d) Spatial distribution of BIM_total_ in Côte d’Ivoire. (e) Spatial distribution of BIM_cocoa_ in Côte d’Ivoire. For easy presentation and comparison, c–e are resampled to a spatial resolution of 100m (area of 1 ha). (f) Spatial distribution of the BF_cocoa_ in each department.

Figure 2a displays the harmonized land use map of Côte d’Ivoire that we developed for this study. It is tailored to meet the needs of biodiversity intactness simulations. This map combines multiple high-resolution local and global earth observations to improve how we identify and categorize agricultural and forest land uses. Figure 2b shows the cocoa cultivation map of Côte d’Ivoire, which differentiates between full-sun and agroforestry cultivation practices. This map uses the most recent remotely sensed cocoa map and detailed local land cover information to provide a more accurate depiction of cocoa cultivation areas compared to other maps. Details on land use and cocoa cultivation in Côte d’Ivoire, as well as comparisons with other maps, are available in Results S1 and S2. Our approach not only ensures that the maps are reliable for model inputs but also offers a template that can be adapted to other regions. This improvement significantly enhances the accuracy of the landscape-scale BIA.

### 3.2 Landscape-Scale Biodiversity Impacts Assessment Uncovers Biodiversity Impact Hotspots

Our assessment framework is spatially explicit at the grid cell level, allowing for the fine-grained identification of local biodiversity impact hotspots. Figure 2c illustrates the spatial distribution of MSA loss, revealing extensive biodiversity intactness losses in most regions of Côte d’Ivoire. Figure S2b maps the spatial distribution of RI, pinpointing the highest biodiversity importance area in Côte d’Ivoire to be Taï National Park and its surroundings. Figure 2d presents the spatial distribution of BIM_total_, highlighting the primary biodiversity impact hotspots located in Côte d’Ivoire’s southeastern and southwestern regions. Remarkably, these regions also surface as hotspots for cocoa cultivation, as evidenced by remotely sensed observations (Figure 2b).

Figure 2e illustrates the spatial distribution of BIM_cocoa_ across Côte d’Ivoire. It shows a pronounced spatial heterogeneity in the biodiversity impacts of cocoa cultivation. The western and central parts of Côte d’Ivoire emerge as areas of critical biodiversity importance with high BIM_cocoa_ values. In contrast, the eastern part of the country shows comparatively low BIM_cooca_ values. For instance, in western Côte d’Ivoire’s forest landscape, biodiversity impacts can be up to 1.8 biodiversity-weighted ha in 1 ha of cocoa cultivation, whereas this value is only about 0.5 in the eastern agricultural landscape. From the overall perspective of Côte d’Ivoire, cocoa cultivation has caused significant biodiversity impacts, accounting for 2.70 × 10^6^ biodiversity-weighted ha (∼10% of the BIM_total_ of Côte d’Ivoire, ∼44% of the BIM_total_ in cocoa cultivation areas). In particular, the biodiversity impacts of cocoa cultivation are most pronounced in the departments of Tabou, Sassandra, and San-Pedro, each exceeding 2 × 10^5^ biodiversity-weighted ha (Figure S14 and Table S8). The abovementioned findings highlight the considerable role of cocoa cultivation in driving biodiversity loss across Côte d’Ivoire.

Figure 2f shows the spatial distribution of BF_cocoa_ in each department. The average BF_cocoa_ in Côte d’Ivoire is 1.25 ha**·**year**·**ton^–1^. The distribution of BF_cocoa_ among departments varies markedly, ranging from 0.5 to 3.2 ha**·**year**·**ton^–1^. Generally, the western departments demonstrate higher BF_cocoa_ compared to the eastern departments. For example, among the top 10 cocoa production departments, the western department of Tabou exhibits the highest BF_cocoa_, nearly 4.5 times greater than that of Abengourou in the east, which has the lowest BF_cocoa_ (as detailed in Table S9).

Figure S13a,b shows the spatial distribution of the PDF of cocoa cultivation and PDF per ton of cocoa produced using the PDF method. Cocoa cultivation in Côte d’Ivoire is predominantly concentrated in two ecoregions: the Western Guinean lowland forests and the Eastern Guinean forests. This method tends to homogenize biodiversity impacts across numerous grid cells within the same ecoregion, as depicted in Figure S13a. Consequently, this homogenization poses challenges in pinpointing biodiversity impact hotspots within ecoregions. Furthermore, the biodiversity impacts per ton of cocoa produced within the same ecoregion are primarily influenced by yield, as shown in Figure S13b. As a complement to this approach, our landscape-scale assessment significantly enhances spatial detail, thereby improving the identification of biodiversity impact hotspots within ecoregions. For instance, Figure S15 demonstrates that the PDF per ton of cocoa produced is remarkably consistent across the top 10 cocoa-producing departments. Conversely, the variability of BF_cocoa_ in each department is distinctly evident.

### 3.3 Role of Export Supply Chains in Driving Biodiversity Impacts

We tracked the tele-connected biodiversity impacts across 6031 cocoa export supply chains in 2019. The biodiversity impacts embodied in these supply chains are 2.50 × 10^6^ ha·year, which amounts to ∼93% of the total biodiversity impacts of cocoa cultivation, of which 1.06 × 10^6^ ha·year (∼42%) can be traced back to specific production departments. The remaining impacts, accounting for ∼7%, are attributable to domestic consumption, processing, and stocks. These results indicate that cocoa exports are the primary driver of biodiversity impacts of cocoa cultivation in Côte d’Ivoire.

**Figure 3:**
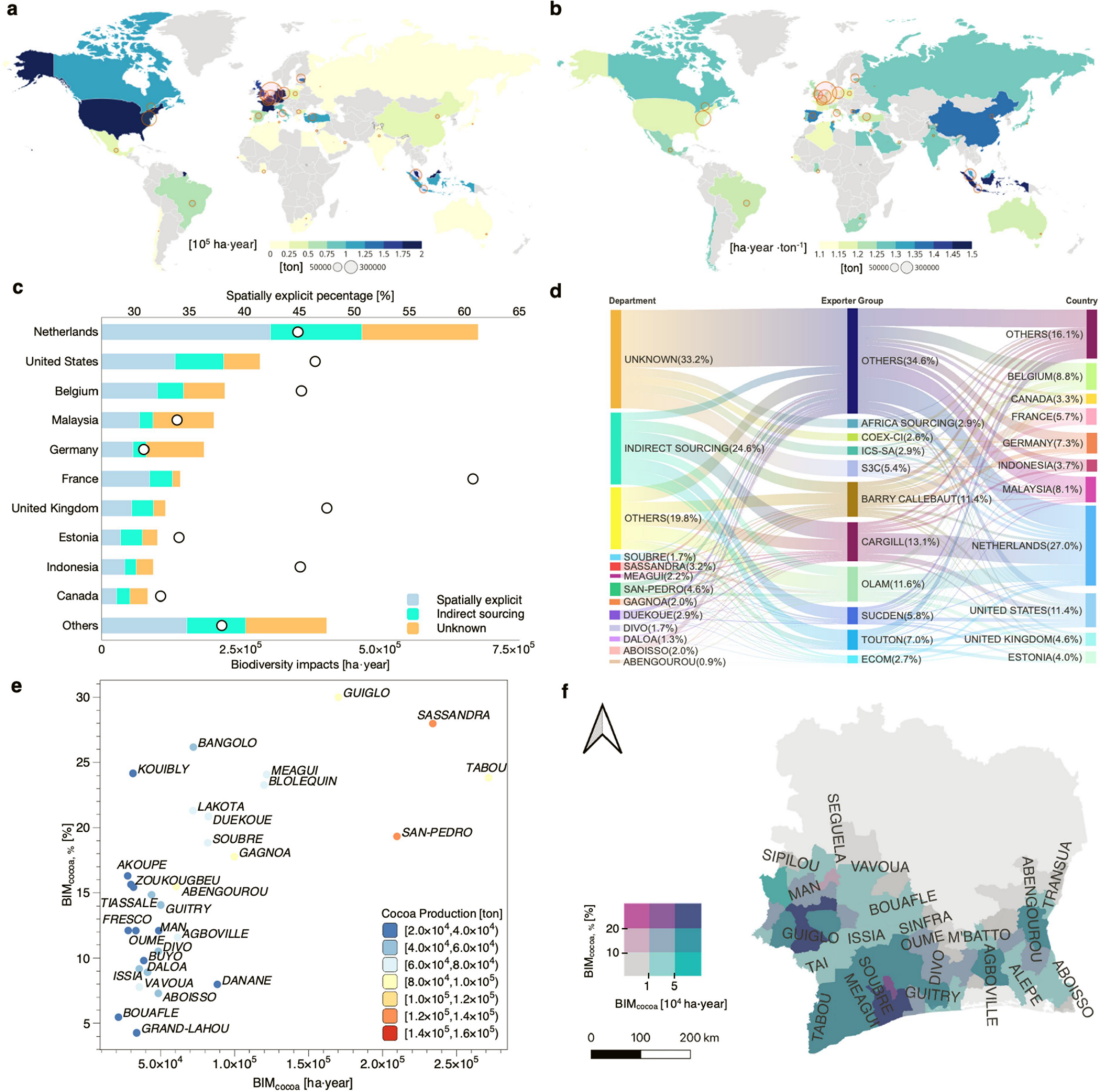
(a) Spatial distribution of the tele-connected biodiversity impacts among importing countries. The size of the red circle indicates the value of the imported mass. (b) Spatial distribution of the biodiversity impacts per ton of cocoa imported among importing countries. (c) Biodiversity impacts statistics for the top ten importing countries with the highest biodiversity impacts. The black hollow circle indicates the percentage of biodiversity impacts that can be tracked to specific production departments. (d) Biodiversity impacts flow along Côte d’Ivoire’s cocoa export supply chain. Production departments are on the figures’ left, exporter groups are in the middle, and importing countries are on the right. The width of the flows is proportional to the total tele-connected biodiversity impacts. (e) Distribution of absolute and relative biodiversity impacts of cocoa cultivation in the top 30 cocoa production departments. The circle color indicates the cocoa production in each department. (f) Bivariate map illustrates the relative spatial distribution of absolute and relative biodiversity impacts of cocoa cultivation in each department.

Figure 3a depicts the tele-connected biodiversity impacts of cocoa cultivation in each importing country. In 2019, cocoa was directly exported to 41 countries, with the top 10 importing countries accounting for ∼84% of the total biodiversity impacts. These leading importers are primarily located in Europe and North America, with Malaysia and Indonesia being the largest importers in Asia. Figure 3b depicts the distribution of biodiversity impacts per ton of cocoa imported among importing countries. Among the top 10 importing countries, Indonesia has the highest spatially explicit BF_cocoa,import_, which is about 34% higher than Germany with the lowest BF_cocoa,import_ (Table S10). The BF_cocoa,import_ differences among importing countries are less significant than the BF_cocoa_ among production departments. That is because importing countries typically source cocoa from diverse export groups which, in turn, draw from various production departments rather than just one (Figure 3d). This diversified sourcing approach limits BF_cocoa, import_ variability among importing countries. Furthermore, the top 10 cocoa export groups, which account for ∼65% of the total export biodiversity impacts, are notable for limited traceability. Nearly 60% of the cocoa volume exported by these groups cannot be traced back to specific production departments (Figure 3d). Africa Sourcing, S3C, ICS-SA, and COEX-CI, all ranking in the top 10 export groups, source their cocoa indirectly, making it untraceable to specific production departments. This underscores the key role of export groups in improving supply chain transparency.

We compared our results with those calculated based on the PDF method across importing countries. Among these countries, Indonesia has the highest PDF per ton of cocoa imported, which is only ∼5% above the lowest value in Poland (Table S10). The assumption of homogeneity within ecoregions serves to further reduce the variability in biodiversity impacts per ton of cocoa imported among the importing countries.

## 4 Discussion

### 4.1 Biodiversity Impacts Comparison Analysis of Full-sun and Agroforestry Cocoa Cultivation

Our landscape-scale assessment framework provides us with the tools necessary for a detailed, regionalized comparison of the full-sun and agroforestry cocoa cultivation systems. In Côte d’Ivoire, full-sun cocoa has a national average BF_cocoa_ that is ∼15% higher than agroforestry cocoa (Figure S15c and Table S11). This indicates that agroforestry cocoa generally has lower biodiversity impacts. We further examined the biodiversity intactness loss (MSA loss) and biodiversity importance (RI) of full-sun and agroforestry cocoa cultivation. On the one hand, MSA loss per ton of agroforestry cocoa produced is ∼18% lower than that of full-sun (Figure S15a and Table S11). This discrepancy may be influenced by the calibration of our model, which systematically assigns higher scores to agroforestry compared to low-input agricultural land use due to its presumed ecological benefits. This trend aligns with existing studies^17,64^ that suggest agroforestry better preserves biodiversity intactness compared to full-sun cocoa cultivation. On the other hand, the production-weighted national average RI of agroforestry systems is 10.0% higher than that of full-sun cocoa (Figure S15b and Table S11), indicating that agroforestry occurs generally in areas with higher endemic species richness. Some of the departments have a lower BF_cocoa_ in full-sun than in agroforestry due to the higher RI associated with agroforestry systems. For example, in the department of Sassandra, although the MSA loss per ton of cocoa produced was ∼24% higher in full-sun than in agroforestry systems, a ∼116% lower RI resulted in a net ∼79% lower BF_cocoa_ for full-sun cocoa. The comparative analysis suggests that agroforestry is not necessarily more biodiversity-friendly than full-sun cocoa, as agroforestry systems tend to be established in areas of higher biodiversity importance (e.g., the establishment of agroforestry systems by partial deforestation in highly biodiverse primary forests). To summarize, in Côte d’Ivoire, agroforestry systems typically have lower biodiversity impacts compared to full-sun cocoa cultivation. Nonetheless, the biodiversity impacts of any cultivation practice, including agroforestry, can be significant in areas with high biodiversity importance. These findings underscore the critical need for a context-specific BIA when implementing cocoa cultivation practices.

### 4.2 Disparity of Absolute and Relative Perspectives on Biodiversity Impacts

We have identified biodiversity impact hotspots in Côte d’Ivoire with absolute perspectives (BIM_cocoa_) and tracked the tele-connected biodiversity impacts along the export supply chain in Section 3. From the relative perspective (BIM_cocoa,%_), cocoa cultivation in 18 departments contributed more than 15% to the department’s total biodiversity impacts (Figure S16b). Across most cocoa production departments, a disparity between absolute and relative perspectives on biodiversity impacts is observed (see Figure 3e,f). For instance, although the BIM_cocoa_ in Kouibly is low, its BIM_cocoa,%_ accounts for ∼24%, signifying the key contribution of cocoa cultivation to the department’s local biodiversity loss. Considering the two main cocoa production departments, Tabou and Guiglo, the absolute biodiversity impacts of cocoa cultivation (BIM_cocoa_) in Tabou are approximately 60% higher than in Guiglo. Conversely, the relative impacts (BIM_cocoa,%)_ in Guiglo substantially exceed those in Tabou, with a notable difference of about 26%. The disparity also exists in the tele-connected biodiversity impacts of importing countries. Using The Netherlands as an example, Table S12 shows each department’s absolute and relative biodiversity impacts of Dutch cocoa imports. Absolutely, the highest impacts of Dutch cocoa imports are concentrated in the Tabou, San-Pedro, and Duékoué departments, indicating that the most significant biodiversity impacts caused by Dutch cocoa imports are in these departments. However, relatively, Dutch cocoa imports have the highest contribution to the biodiversity loss in Duékoué, Yakassé-Attobrou, and Djékanou departments. Thus, in these departments, Dutch cocoa imports result in a higher pressure on their local biodiversity relative to other types of biodiversity pressures. These findings have significant implications for developing more sustainable cocoa cultivation and formulating more comprehensive policies for biodiversity conservation.

## 5 Limitations and Outlook

We have developed a harmonized land use map for Côte d’Ivoire with a spatial resolution of ∼10 m. This map was integrated with landscape-scale biodiversity models to assess the land use-related biodiversity impacts of cocoa cultivation and trace biodiversity impacts along the export supply chains. However, this study comes with several limitations:

(1) Validating the accuracy of the land use map is challenging due to insufficient field sampling data and the high amount of data points (∼4 billion grid cells). We depended on the integration of numerous state-of-the-art, fine-grained data sets for exceptional spatial detail and compared them with satellite true-color observations for visual check. (2) To maximize the integration of available spatial data, we assumed a static land use status from 2015 to 2020, which ignores land use changes. (3) Our biodiversity intactness model does not comprehensively understand the biodiversity response to landscape pressures. For instance, Outhwaite et al.^65^ found that different landscape-scale variables can affect community total abundance or species richness (e.g., distance to the forest and the number of land-cover types in the landscape), which is not explicitly considered in the GLOBIO-InVEST model. (4) The pressure-effect parameters used in GLOBIO-InVEST are derived from different studies or expert perspectives. The inconsistency of these parameters may lead to errors in the results. Additionally, cocoa cultivation is classified in this study as a general agricultural activity, which may overestimate or underestimate its biodiversity impacts. In future research, the use of crop-specific pressure-effect parameter settings will help to characterize the biodiversity impacts more accurately (e.g., BII responses to cocoa cultivation areas^64^). (5) We use the range rarity variants to measure the contextual biodiversity importance. However, the possible errors in the range of each species cannot be quantified, and whether range rarity and current land use status are independent of each other remains to be investigated. (6) This study uses detailed earth observation data sets, such as local land cover and cocoa cultivation maps, which are often unavailable. As high-resolution mapping technology advances, the applicability of our methodology will improve. In the absence of local maps, global maps can also be effectively integrated into our framework. (7) This study only considers the direct biodiversity impacts of land occupation for cocoa cultivation and does not consider indirect impacts (such as disturbances to biodiversity caused by road construction for cocoa expansion). (8) Our study employs subnational supply chain data to trace tele-connected biodiversity impacts. However, it is important to note that the availability of this level of detailed data is currently restricted to a limited number of commodities and producing countries. Consequently, this limitation restricts the broader applicability of our findings. (9) Only >40% of cocoa exports can be traced back to the production departments, and our approach has not tracked the biodiversity footprint at the consumption stage.

To enhance the study’s methodology, we propose the following future research directions: (1) Refinement of land use allocation frameworks, such as implementing machine learning-driven approaches and detailed land use map benchmarking using field survey data. More detailed land use maps facilitate better assessment of land use-related biodiversity impacts. (2) We suggest the development of landscape-scale biodiversity models that are suitable for high-resolution land use maps and consider more spatial or taxa details in the representation of biodiversity. For instance, the latest GLOBIO4^12^ model calculates MSA responses for different taxa (e.g., plants, birds, and mammals), and the GLOBIO-Species^66^ model computes species-level biodiversity responses that could serve as a starting point. (3) Although this study concentrates on current land use, the model could be adapted to assess the biodiversity impacts of varying scenarios and historical land use dynamics through the creation of land use transformation models. (4) We advocate for increased transparency and availability of spatially explicit supply chains for key agricultural food commodities, a step that would broaden the applicability of our framework. To further enrich our analysis, more detailed supply chain data sets to include final consumption stages can be obtained by integrating Trase data sets with MRIO tables (e.g., Green et al.^9^ traced biodiversity impacts of Brazilian soy production to global drivers), methods^67,68^ based on bidirectional trade data sets, or a combination thereof^69,70^ to trace the biodiversity footprint more comprehensively.

This study can help policymakers, agri-food producers, and consumers to make informed decisions for sustainable agri-food production and consumption. Our findings suggest that effectively mitigating the biodiversity impacts of cocoa cultivation in Côte d’Ivoire will require the implementation of tailored regional land use regulations and the promotion of agroforestry practices. In addition, the establishment of a transparent traceability system for cocoa export supply chains, along with robust biodiversity monitoring and sustainability regulations for stakeholders (e.g., export groups), can further strengthen the outcome of conservation efforts. Our adaptable framework can be extended to other crops and countries and is relevant to addressing other environmental impacts, such as land use-related carbon emissions, or different sectors, such as mining.

## Data Availability

All codes and data sets created in this paper will be publicly available through a Github repository and Zenodo once the paper is accepted.

## Supporting Information Available

Mapping a harmonized land use map for Côte d’Ivoire, GLOBIO-InVEST modeling, comparative analysis, comparison of land use maps, comparison of cocoa cultivation maps, uncertainty analysis, and additional figures and tables (PDF)
