# Supplementary material for: Landscape-Scale Biodiversity Impacts Analysis of Côte d’Ivoire’s Cocoa Cultivation along Export Supply Chains
Source: Environ Sci Technol. 2024 May 18;58(22):9601–11. doi: 10.1021/acs.est.3c07795 (PMC11155242; doi:10.1021/acs.est.3c07795)
Supplement: Supplementary file 1 — es3c07795_si_001.pdf [file es3c07795_si_001.pdf]

Supporting Information:

A Landscape-scale Biodiversity Impacts Analysis of Côte d'Ivoire's Cocoa Cultivation Along Export Supply Chains

Shuntian Wang [1, 2, *], *Stephan Pfister* [1, 2]

1. Department of Civil, Environmental and Geomatic Engineering, Institute of Environmental Engineering, Ecological Systems Design, Swiss Federal Institute of Technology, ETH Zurich, 8093 Zurich, Switzerland;

2. Department of Humanities, Social, and Political Sciences, Institute of Science, Technology, and Policy (ISTP), Swiss Federal Institute of Technology, ETH Zurich, 8092 Zurich, Switzerland.

*Corresponding author: shuntian.wang@ifu.baug.ethz.ch

This file includes:

Number of pages: 43

Number of methods sections: 3

Number of results sections: 3

Number of figures: 16

Number of tables: 13

Supporting references

Supplementary methods

Methods S1: Mapping a harmonized land use map for Côte d'Ivoire

Pre-processing of input datasets:

We constructed a harmonized land use map of Côte d'Ivoire by integrating 7 raster, vector, and numerical datasets based on remote sensing, and national or regional statistics. To enable the integration of these datasets, we preprocessed the data for consistency. First, all spatially explicit data layers were clipped to the spatial extent of Côte d'Ivoire. The Côte d'Ivoire border vector data are available on the open-source site GADM[1]. We then use the nearest neighbor method to resample the raster or vectors to the spatial extension of the BNETD 2016 map with a spatial resolution of ~10m.

Agriculture allocation:

As our point of interest was the land-use-related biodiversity impacts of cocoa cultivation, we give the highest priority to cocoa grid cells and differentiated cocoa cultivation types. Cocoa cultivation types include full-sun cocoa and agroforestry cocoa. Full-sun cocoa, also known as monoculture cocoa, is typically grown in large, open fields where the trees are exposed to full sunlight. Agroforestry cocoa is grown more diversely and sustainably. In agroforestry systems, cocoa trees are intercropped with other plant species, such as shade trees and fruit trees. We differentiate cocoa cultivation types by integrating cocoa map and BNETD 2016 map. Cocoa grid cells identified as ‘Agroforestry management’ and ‘Degraded agroforestry management’ in BNETD 2016 map are allocated to agroforestry cocoa, while other cocoa grid cells are allocated to full-sun cocoa.

We allocated the other agricultural grid cells from BNETD 2016 map. The grid cells labeled as ‘Coffee-cocoa’, ‘Rubber tree’, ‘Coconut palm’, and ‘Cashew tree’ were allocated to ‘Low-input agriculture’.

The grid cells classified as ‘Agroforestry management’ and ‘Degraded agroforestry management’ were allocated to ‘Agroforestry’. The agriculture grid cells in Côte d'Ivoire were not classified as ‘High-input agriculture’ because we used the GLOBIO4[2] methodology to calculate land use intensity. Based on the results, all agriculture grid cells in the country are considered low input. The results is also consistent with existing research’s assumptions[3].

Pasture allocation:

We took the average value of the FAOSTAT land use statistics for ‘Land under temp. meadows and pastures’ and ‘Land under perm. meadows and pastures from 2015 to 2020 as the total pasture area. We allocated pasture grid cells according to their suitability values and the total pasture area from the grid cells with the highest suitability until the total area was allocated. Tropical Livestock Units (TLUs) are a way of standardizing and comparing different types of livestock based on their weight and size in tropical regions. We first computed each grid cell’s total TLUs based on the density of ruminants (cattle, goat, sheep) in the Gridded Livestock of the World database (GLW v4)[4] datasets. The conversion factors we used are one goat or sheep = 0.1 TLUs and one cattle = 0.6 TLUs[5]. We specified that pasture could only be allocated to grid cells categorized as ‘Wooded savannah’, ‘Shrub formation’, and ‘Herbaceous formation’ from BNETD 2016 map. We utilized Potential Natural Vegetation (PNV) map to differentiate between ‘Man-made pastures’ and ‘Livestock grazing’. Pasture grid cells with herbaceous/shrub vegetation PNV are categorized as ‘Livestock grazing’. However, since all allocated pasture grid cells had tree PNV, we classified all pasture grid cells as ‘Man-made pastures’.

Forest allocation:

We allocated the remaining forest grid cells in BNETD 2016 map to different types of forests. We used the ~100m spatial resolution Global Forest Management Data (GFMD)[6] map to obtain the spatial

distribution of primary and plantation forests. GFMD map divides the other forest grid cells into naturally regenerating forests with signs of human activities, oil palm plantations, and agroforestry. We did not use these three forest types for allocation because agroforestry and oil palm plantations have been considered in agricultural land use. We used the Forest Landscape Integrity Index (FLII)[7] for further allocation. FLII is a globally consistent, continuous index of forest condition as determined by the degree of anthropogenic modification by integrating data on observed and inferred human pressures and the index of lost connectivity. We allocated the remaining forest grid cells with FLII larger than 0.6 as 'Lightly used natural forest' and the FLII smaller than 0.6 as 'Secondary forest', corresponding to medium integrity and low integrity in the original paper. The remaining unallocated grid cells are allocated to 'primary vegetation', which is mostly present in protected areas.

Methods S2: GLOBIO-InVEST modeling

The GLOBIO model provides a biodiversity intactness index based on mean species abundance (MSA), which is the average response of populations of different species to various stressors such as land use changes, fragmentation, and infrastructure[8], MSA ranges from 0 to 1, where 1 means the species composition is completely intact, and 0 means that all original species are extirpated (locally extinct). The GLOBIO-InVEST model extends the GLOBIO3 method to downscale their global approach to the landscape level to identify finer ecological responses that may include nonlinearities[9]. The main differences between GLOBIO-InVEST and GLOBIO3 are that GLOBIO-InVEST uses a more sophisticated approach to quantifying fragmentation than applying overall averages of patch size in different habitats. GLOBIO-InVEST replaces the standard GLOBIO3 model with fragmentation analysis using a fragmented forest quality index (FFQI). The FFQI is calculated by considering how many of a forest's neighboring cells are also forested. GLOBIO-InVEST MSA simulation is based on the same cause-effect philosophy as GLOBIO3. The details of the cause-effect parameters we used in

this study are in Table S3. The parameters are based on a broad literature review and suggested methodologies for inputting and processing the spatial data required. During the simulation, suppose the land use type of grid cell i is lu, the proximity of infrastructure is *I*, and FFQI is *F*. The MSA for grid cell i is calculated by multiplicative as follows：

$$MSA_i = MSA_{lu_i} \cdot MSA_{I_i} \cdot MSA_{F_i} \quad (1)$$

We assumed that the cause-effect parameters satisfy a normal distribution to obtain the upper and lower bounds for the MSA simulations. We used the mean parameters plus or minus two times its standard deviation to determine the MSA distribution with 95% confidence intervals. The range of the MSA cause-effect parameters are limited from 0 to 1.

Methods S3: Comparative Analysis

We compared the results of the BIM method with the PDF method to illustrate the perspective brought by our landscape-scale assessment approach. PDF accounts for a fraction of species richness potentially lost due to an environmental mechanism. We employed the UNEP life cycle initiative recommended CFs for PDF calculation. The CFs were generated using the countryside species−area relationship (c-SAR) and vulnerability scores to estimate PDF per unit area of land occupation/transformation in 804 terrestrial ecoregions across five taxa and six land use types[10]. We only considered the land occupation of cocoa cultivation, and the CFs we used for the different ecoregions are shown in Table S4, and the spatial distribution of ecoregions in Côte d'Ivoire is shown in Figure S7. We first rasterized the ecoregion vector files to the spatial extension of the satellite remotely sensed cocoa map. We then allocated CFs based on the ecoregion corresponding to each cocoa grid cell. Finally, we multiplied the CFs of each cocoa grid cell with the grid cell area to obtain the global PDF of each cocoa grid cell. We

calculated the PDF per ton of cocoa produced and linked PDF results to the export supply chains in the same way as in sections 2.2.3 and 2.3.

Supplementary Results

Results S1: Comparison of land use maps

In our harmonized land use map, an area of 11,222 kha has been allocated to agricultural land, which is ~12% larger than the figure reported by FAOSTAT. This includes 8,207 kha of low-input agricultural land and 3,015 kha of agroforestry land. An area of 3,890 kha has been allocated to forest, which is ~32% larger than the FAOSTAT figure and ~30% larger than the figure of the National Forest and Wildlife Inventory (IFFN) of Côte d'Ivoire[11]. This discrepancy is due to different definitions of forest as well as temporal inconsistency. In the harmonized land use map, secondary forest dominates the forest land, covering 2,251 kha, with lightly used natural forest coming next at 1,134 kha. Primary and plantation forests are seen to occupy a relatively small area. An allocation of 13,604 kha has been made to man-made pastures, a figure that maintains consistency with the FAOSTAT data since we use FAOSTAT data as the total area reference for pasture.

We compared our harmonized land use map to GLOBIO4[2] (Figure S5) and HILDA+[12] (Figure S6). There is a clear discrepancy between the GLOBIO4 and HILDA+ land use maps in southern Côte d'Ivoire. GLOBIO4 typically classifies most of the grid cells in this region as agricultural land, while HILDA+ classifies them as forest. Therefore, instead of grouping tree grid cells into a single category, our map effectively distinguishes perennial tree crops and agroforestry from tree cover by integrating detailed local land cover maps and allocation rules. Comparatively, the total agricultural and forest land allocation of our map lies between the results of GLOBIO4 and HILDA+ and is closer to the FAOSTAT reporting data (Figure S9).

Through visual inspection using Sentinel-2 cloudless 2020 and the high-resolution land cover map (WorldCover 2020), we further evaluated these land use maps in typical landscapes (Figure S8). The

visual inspection shows that our harmonized land use map provides more detailed spatial granularity and landscape information than both GLOBIO4 and HILDA+.

The discrepancies between existing land use maps and the precision offered by our approach underscore the complexity and dynamic nature of land use classification. Moreover, our approach demonstrates the importance of integrating multiple data sources and validation techniques to produce more reliable land use maps. It underscores the utility of detailed support layers and locally sourced land cover data in refining land use datasets.

Results S2: Comparison of cocoa cultivation maps

The total remotely sensed cocoa cultivation area was 4,450 kha, ~0.5% less than the FAOSTAT figure. Agroforestry cocoa accounted for ~30% of the total cocoa cultivation area. The cocoa cultivation area aligns well with climatically suitable growing regions[13] and dominates land use in these regions. In the cocoa climatically suitable growing regions, over 26% of the land is used for cocoa cultivation (Figure 2b and S10). In Bas-Sassandra, cocoa cultivation even accounts for ~42% of its total land area.

We compared the remotely sensed cocoa cultivation map used in this study with the Spatial Production Allocation Model (SPAM)[14] and another remotely sensed cocoa map (referred as UoW)[15]. To facilitate comparison, we resampled both remotely sensed cocoa distribution maps to the spatial extent of SPAM. First, there is a significant difference in the cocoa cultivation maps. The difference of the cocoa cultivation area can vary by four times in the same region (e.g., Bas-Sassandra). Second, the cocoa distribution in both remotely sensed maps was more concentrated in the southwest and southeast regions of Côte d'Ivoire, which corresponds well with the climatically suitable growing regions[13], while the SPAM cocoa map showed a more dispersed distribution (Figure S11). This dispersed distribution caused its area in cocoa climatically suitable growing regions being much lower than the remotely

sensed maps. This discrepancy will prevent us from correctly identifying the biodiversity impact hotspots of cocoa cultivation.

We further verified the cocoa cultivation map by assessing the consistency between cocoa production estimated based on cocoa map and cocoa production data from Trase supply chain. For the convenience of comparison, we first aggregated the cocoa production into each department. The production estimates from the remotely sensed cocoa map showed lower values than Trase records export volume in only 5 departments. The total negative deviation is approximately 12,303 tons. On the other hand, SPAM produced similar results in 14 departments, showing a total negative deviation about 18 times higher than that based on remotely sensed cocoa maps. We also tested the correlation of cocoa production of the spatially explicit part of the Trase dataset with production estimates from the remotely sensed cocoa map (Figure S12a) and with SPAM (Figure S12b). We found that the relation with remotely sensed cocoa production estimates (R = 0.81) is higher than with SPAM (R = 0.48). The above comparisons suggest that the remotely sensed cocoa map provides a more accurate representation of cocoa cultivation in Côte d'Ivoire and is more consistent with Trase supply chain data.

Results S3: Uncertainty analysis

In Côte d'Ivoire, we found that the average relative errors of the biodiversity impacts of cocoa cultivation from using different land use maps (specifically HILDA+ and GLOBIO4) and different cause-effect parameters varied significantly, ranging from -62% to 101%. This error is much smaller than the PDF method (the average relative error of the PDF method is more than 400%). We performed further checks, and the relative error does not have a clear impact on the identification of biodiversity impact hotspots and our other results. We have detailed the upper and lower bounds/ relative errors of the biodiversity impacts of cocoa cultivation in each cocoa production department in Table S8. The

upper and lower bounds of the biodiversity impacts per ton of cocoa produced in each cocoa production department are detailed in Table S9. In addition, the upper and lower bounds of the tele-connected biodiversity impacts of cocoa imports in different importing countries are listed in Table S13.

Supplementary Figures

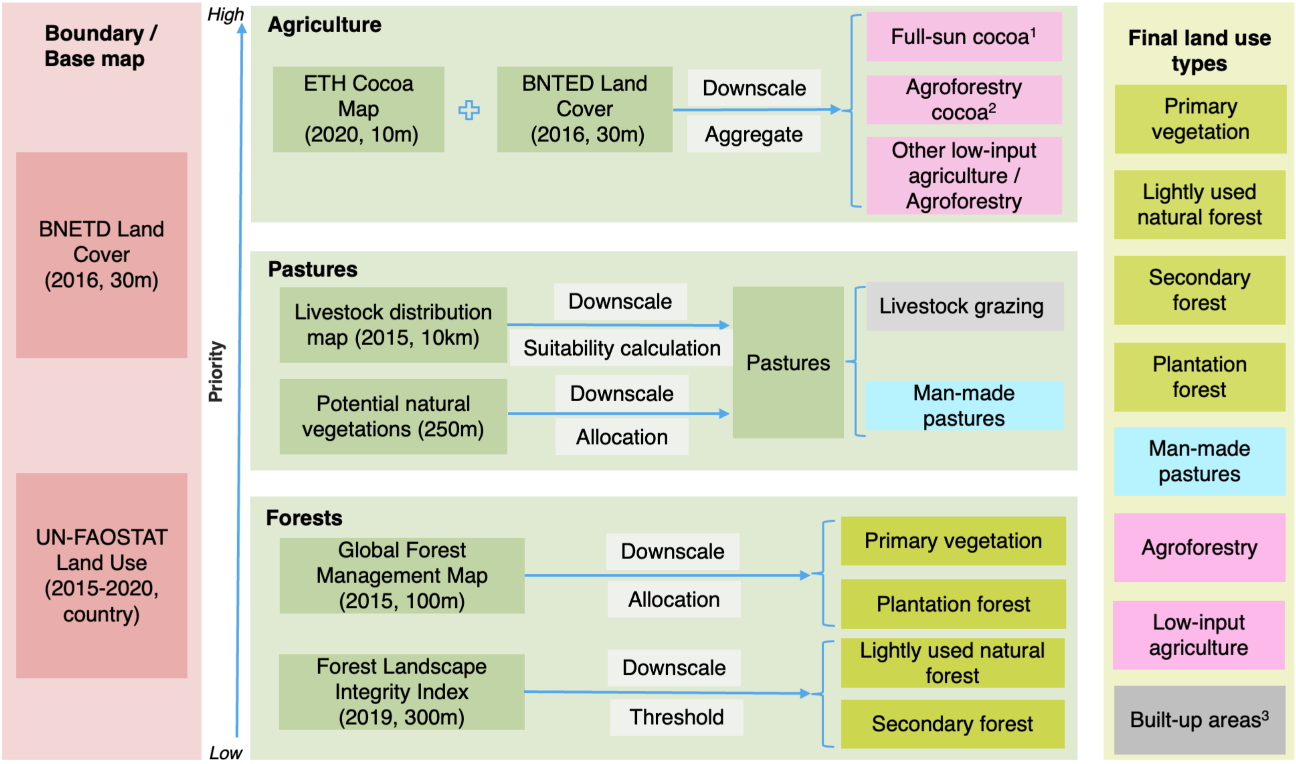

1. 'Full-sun cocoa' is integrated into 'Low-input agriculture'; 2. 'Agroforestry cocoa' is integrated into 'Agroforestry'; 3. 'Built-up areas' are directly obtained from the BNETD map.

Figure S1. The flow chart for constructing the harmonized land use map of Côte d'Ivoire.

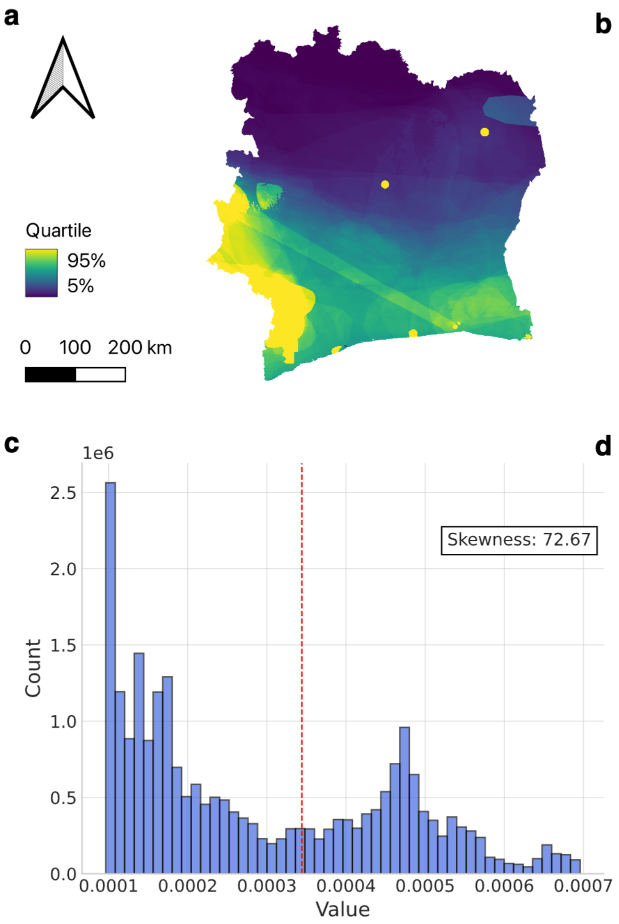

Figure S2. (a) The spatial distribution of the aggregated rarity-weighted richness. (b) The spatial distribution of the rarity index (RI). (c) Histogram of the frequency distribution of the aggregated rarity-weighted richness. (d) Histogram of the frequency distribution of RI.

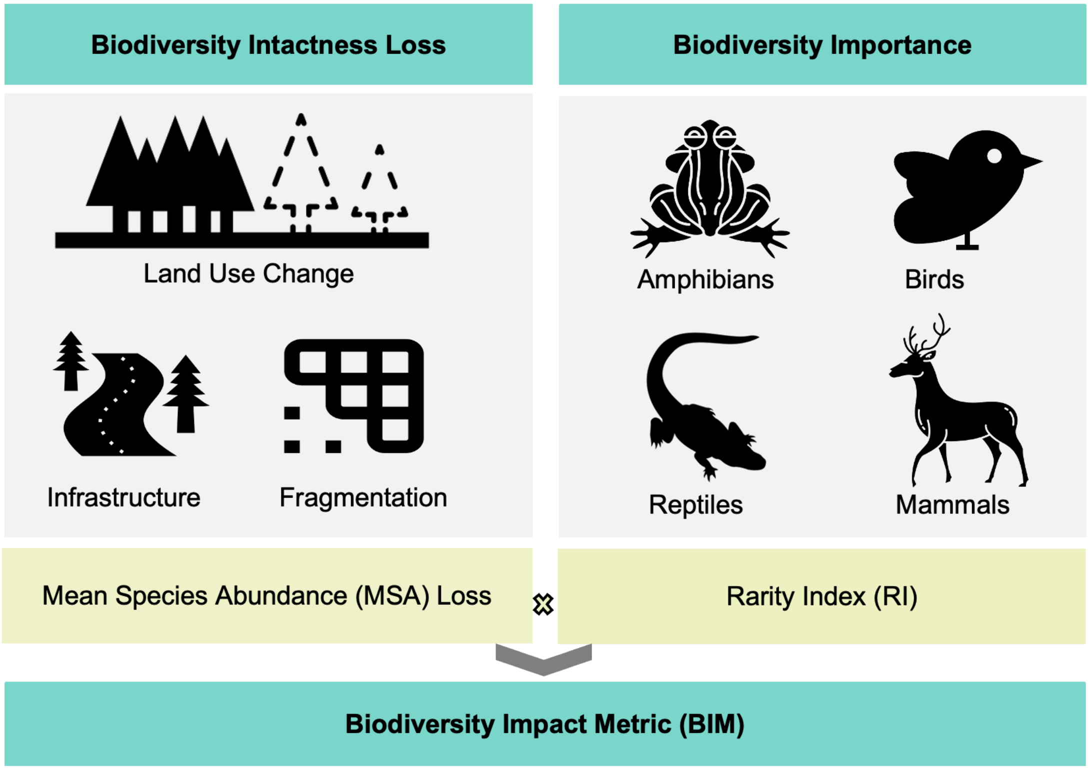

Figure S3. Diagrammatic representation of the biodiversity indicators utilized in this research.

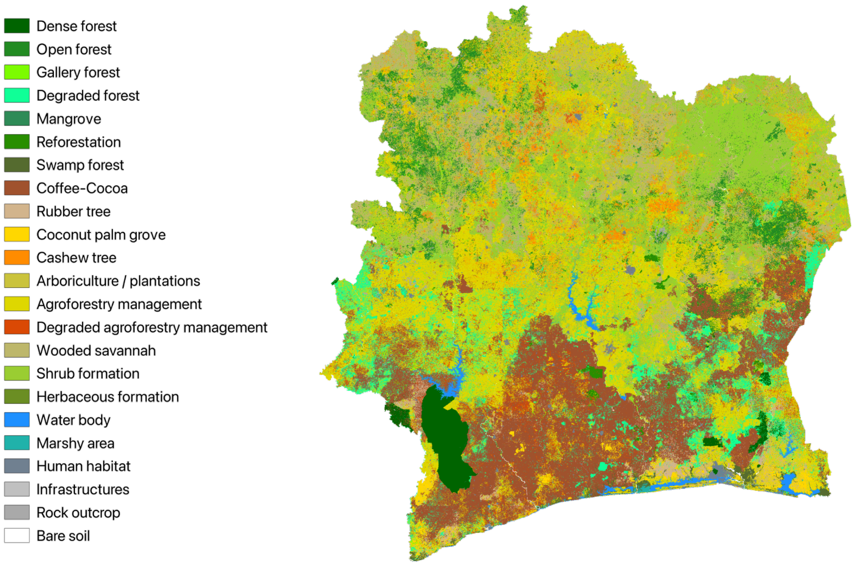

Figure S4. The BNETD 2016 land cover map used to create the harmonized land use map in this study.

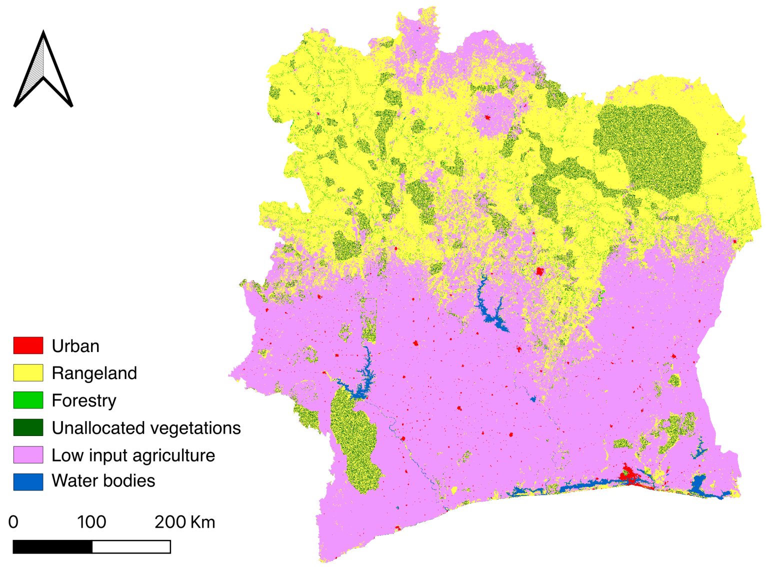

Figure S5. The GLOBIO 4 land use map (2015, 300m). Forest or shrub grid cells that are not allocated to human activity land use types are integrated as 'Unallocated vegetations'.

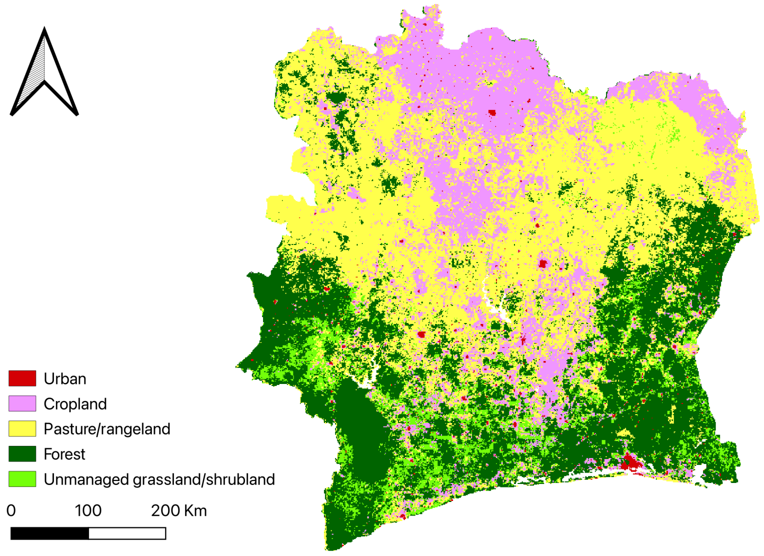

Figure S6. The HILDA+ land use map (2019, 1km).

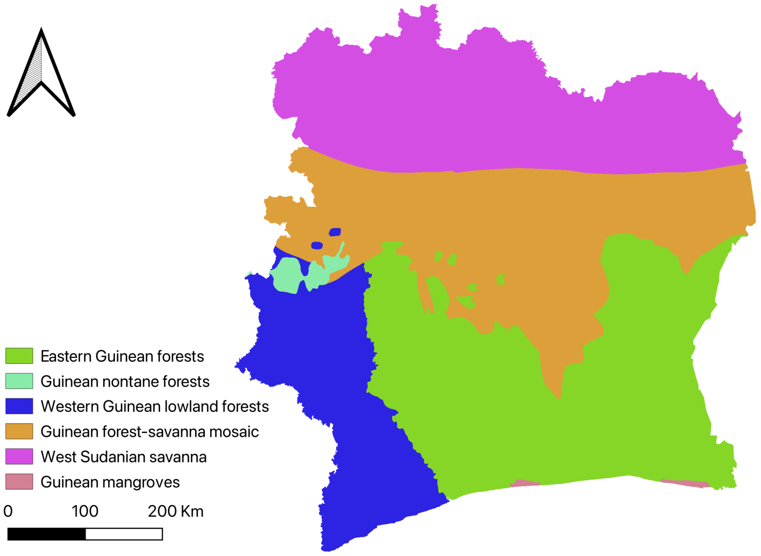

Figure S7. Spatial distribution of ecoregions in Côte d'Ivoire.

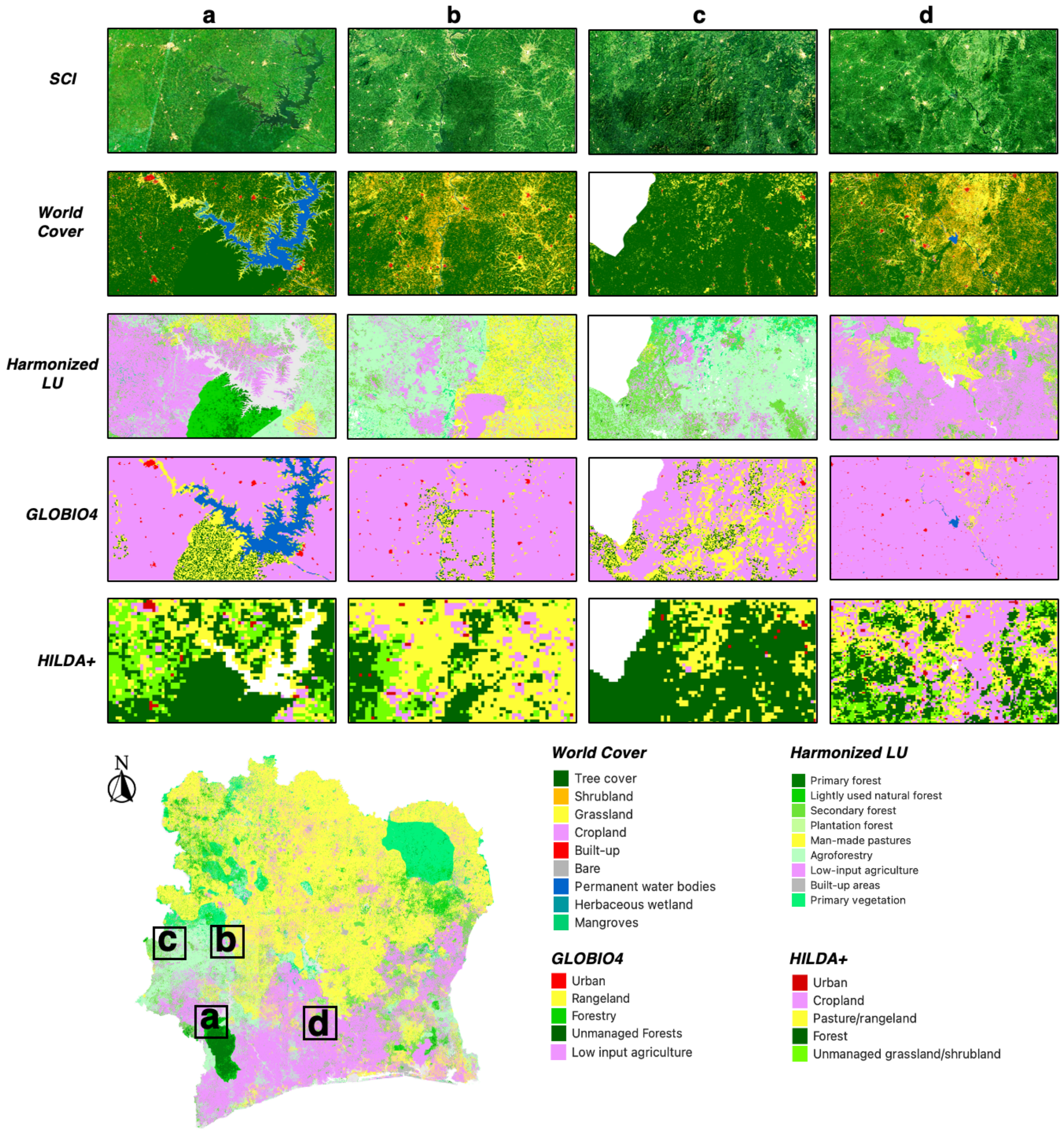

Figure S8. Comparisons of harmonized land use map (Harmonized LU), Sentinel-2 cloudless image (SCI), high resolution land cover map (ESA WorldCover 2020), GLOBIO4, and HILDA+ in typical Landscapes. The central coordinates of the selected landscapes are a (6.3829° N, 7.2706° W), b (7.3674° N, 6.9974° W), c (7.6013° N, 7.9166° W), and d (5.8710° N, 4.4380° W).

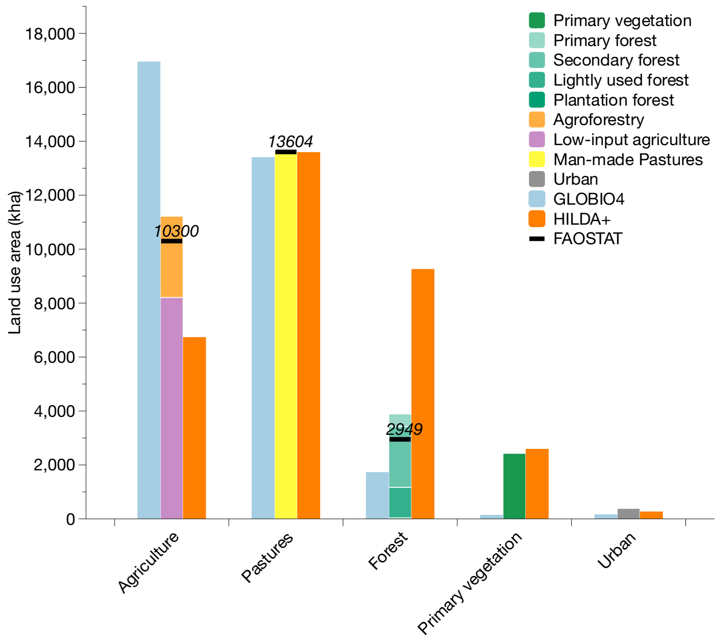

Figure S9. Area Statistics of land use in Côte d'Ivoire, with the harmonized land use map for this study represented by stacked bars, and HILDA+ and GLOBIO4 by a single bar, the data reported by FAOSTAT are represented by black horizontal lines and numbers.

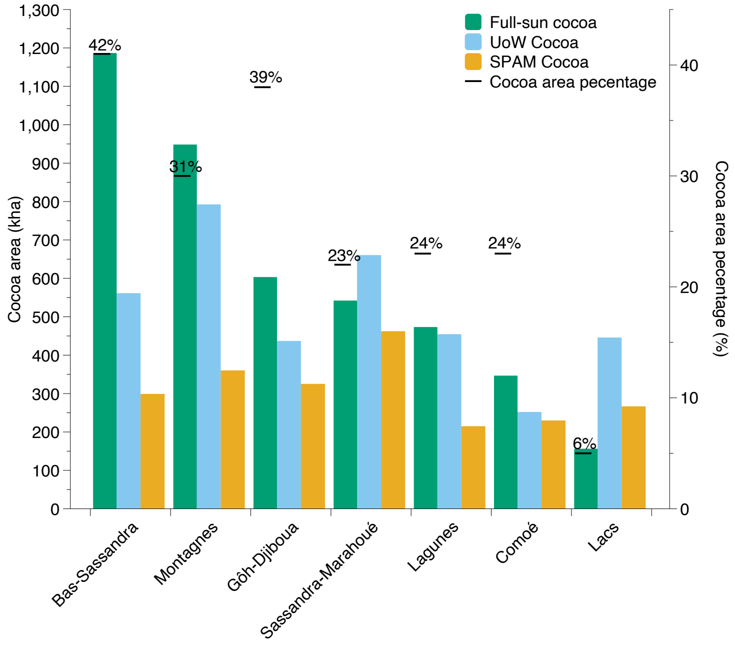

Figure S10. Area statistics of different cocoa maps across climatically suitable growing regions, with black horizontal lines indicating the percentage of the region's total land area under cocoa cultivation.

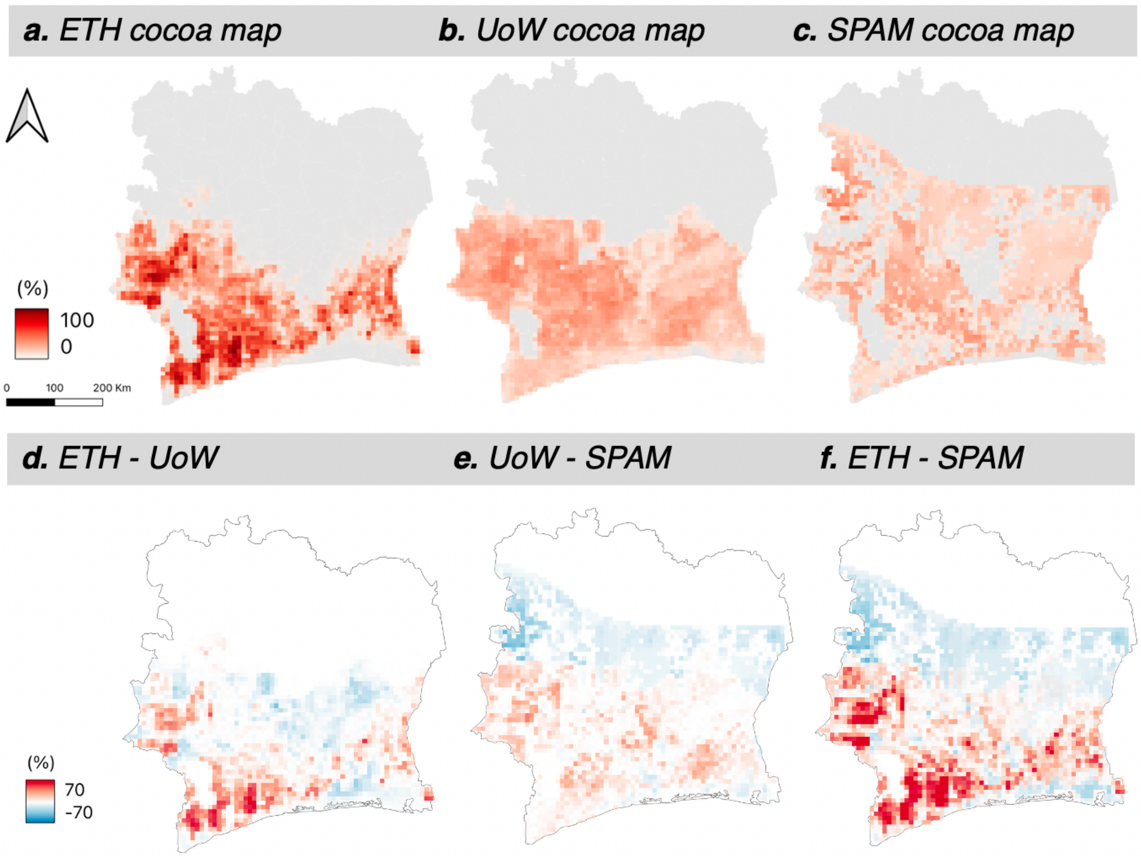

Figure S11. Comparisons of the cocoa cultivation maps. (a) remotely sensed cocoa map[16] used in this study, (b) remotely sensed UoW cocoa map[15] and (c) SPAM cocoa harvested area map[14]. The cocoa maps were all resampled to the SPAM grid cells for easier comparison. d-f shows the relative differences between the different cocoa maps.

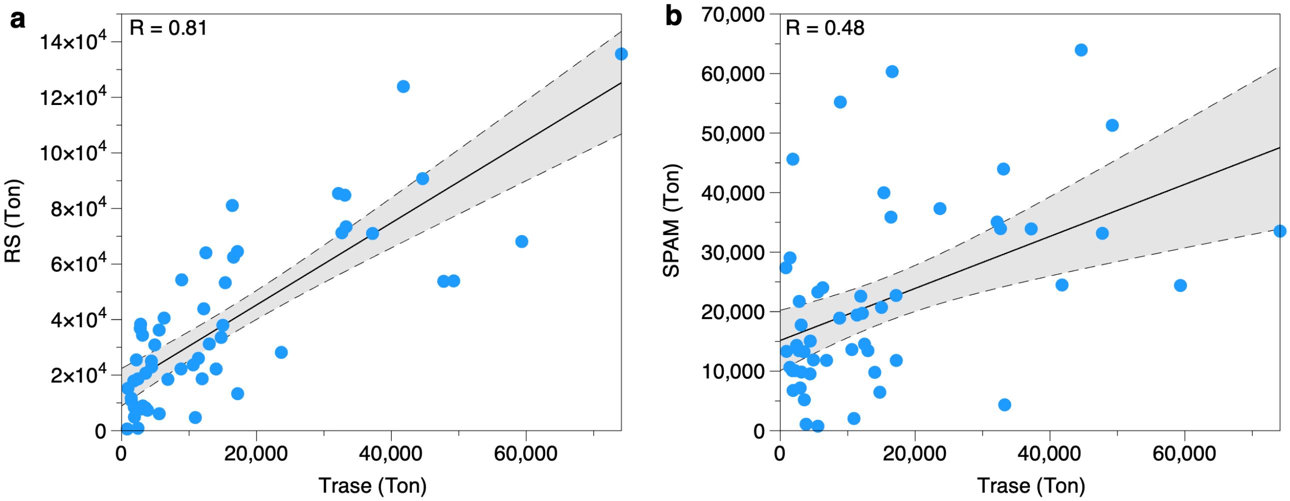

Figure S12. Correlations between the cocoa production estimates with Trase export volume at each department. (a) Correlation graph between cocoa production estimates based on remotely sensed (RS) cocoa map and Trase export volumes; (b) Correlation graph between SPAM cocoa production estimates and Trase export volumes.

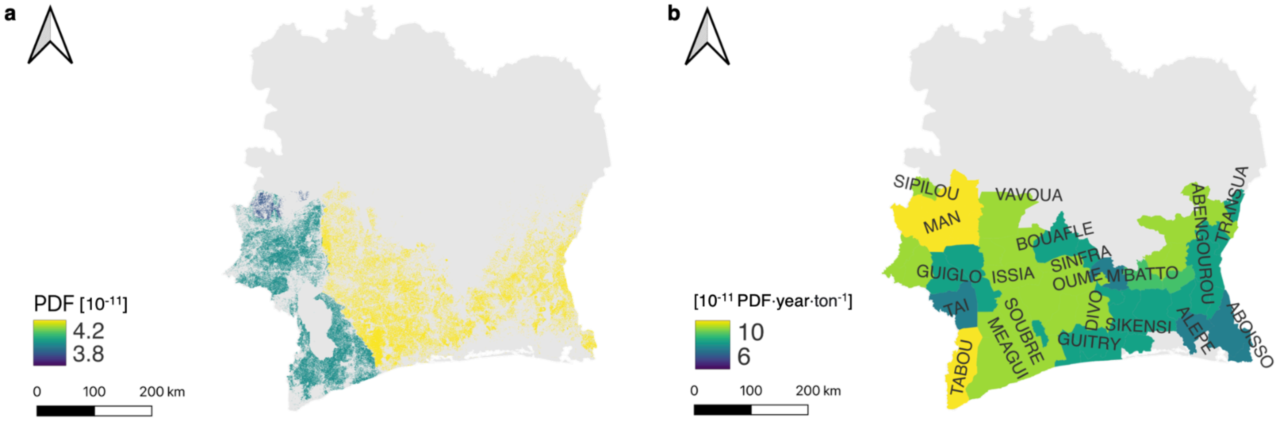

Figure S13. (a) the spatial distribution of the PDF of cocoa cultivation. (b) the PDF per ton of cocoa produced in each department.

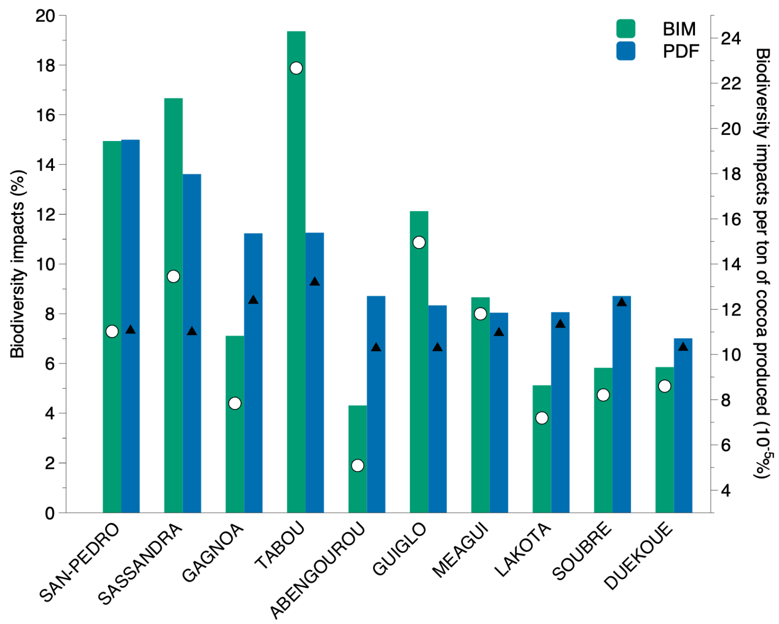

Figure S14. Comparative analysis of biodiversity impacts and biodiversity impacts per ton of cocoa produced in the top 10 cocoa production departments. Biodiversity impacts, or biodiversity impacts per ton of cocoa produced, is presented as the relative percentage of the total biodiversity impacts due to cocoa cultivation in Côte d'Ivoire for easier comparison. The bar chart is used to represent the relative biodiversity impacts of each department. The circle represents the relative BIM per ton of cocoa produced by each department, and the triangle represents the relative PDF per ton of cocoa produced by each department.

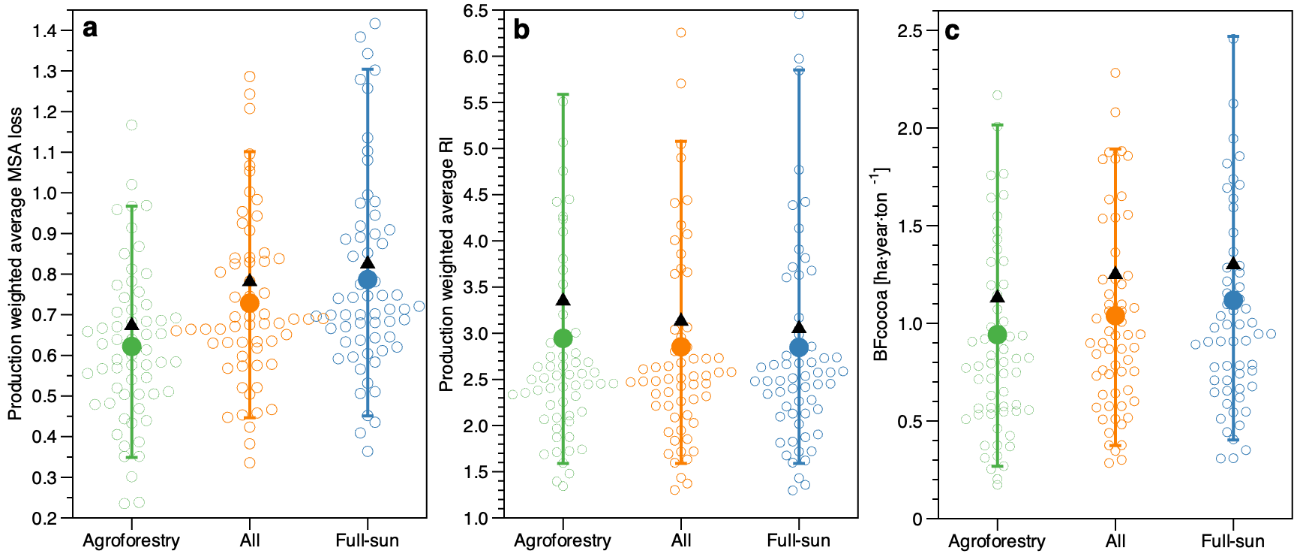

Figure S15. Panels a-c display box-and-whisker plots, illustrating the biodiversity indicators for each department with production volumes surpassing 1000 tons. The biodiversity indicators are as follows: (a) production weighted average MSA loss; (d) production-weighted average RI; (e) BF_{cocoa}. The box-and-whisker plots' terminal ends denote 95% confidence intervals, while the filled circles symbolize the median of the biodiversity indicator. Solid black triangles denote the mean biodiversity indicator. The dashed circles represent the indicators of each department.

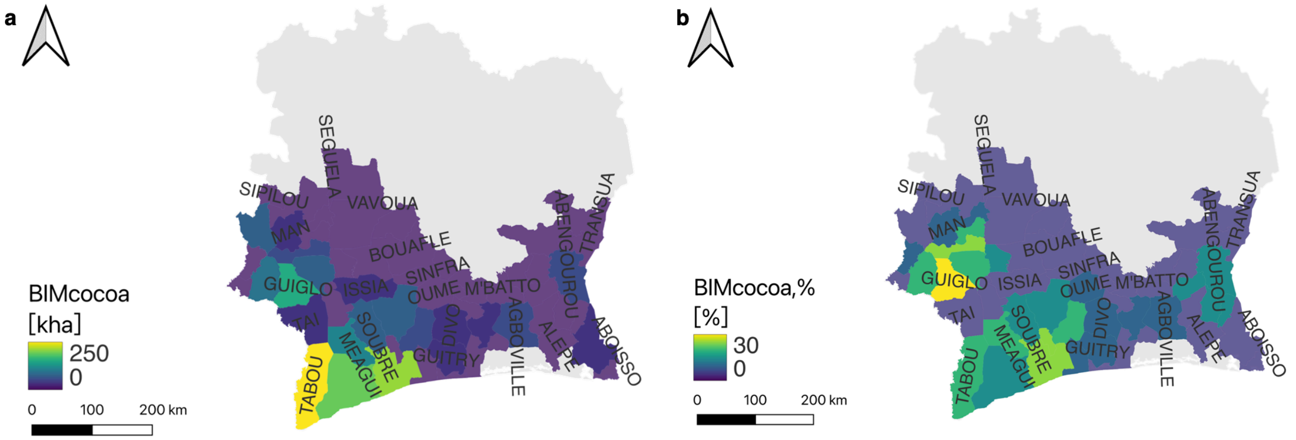

Figure S16. (a) the BIM_{cocoa} in each department. (b) the $BIM_{cocoa, \%}$ in each department.

Supplementary Tables

Table S1. Definition of land use types in this study.

**Land use category**		
Value	Name	Definition
**1**	Primary forest	Naturally regenerated forests of native tree species, where there are no clearly visible indications of human activities, and the ecological processes are not significantly disturbed.
**2**	Lightly used natural forest	A forest that has been subjected to some level of human activity but still retains many of its natural characteristics and ecological functions.
**3**	Secondary forest	A forest that has re-grown after a timber harvest or clearing for agriculture, until a long enough period has passed so that the effects of the disturbance are no longer evident.
**4**	Plantation forest	A forest that has been deliberately planted and managed to produce timber, pulp, or other forest products.
**6**	Man-made pastures	The pastures are created by removing natural vegetation and then sowing grasses or other forage crops that are suitable for animal grazing.
**7**	Agroforestry	Agroforestry is a land use type that integrates the cultivation of trees or shrubs with crops or livestock on the same piece of land. The ‘agroforestry’ in this study was limited to land use for cocoa cultivation under the canopy shade above 15m.
**8**	Low-input agriculture	Low-input agriculture is a land use type of farming that relies on minimal external inputs, such as fertilizers, pesticides, and energy, and instead emphasizes the use of natural resources and ecological processes to maintain soil fertility and manage pests and diseases.
**10**	Built-up areas	The presence of buildings (roofed structures). Includes paved surfaces (roads, parking lots), commercial and industrial sites (ports, landfills, quarries, runways), and urban green spaces (parks, gardens).
**21**[*]	Primary vegetation	The plant communities (excluding forest) that have developed without significant human intervention or disturbance, such as clearing or planting.

[*] In the biodiversity intactness modeling, ‘Primary forest’ and ‘Primary vegetation’ were combined into one land use type.

Table S2. The input datasets used to construct the land use map of Côte d'Ivoire.

Input datasets category						
No.	Name	Time	Resolution	Description	Access	Usages
**1**	BNETD Land Cover Map	2016	30m	BNETD provides detailed land use and land cover types for the entire territory of Côte d'Ivoire based on Earth observation and field sampling data.	Reasonable request to the National Bureau of Technical and Development Studies in Côte d'Ivoire.	Base map
**2**	ETH Cocoa Map	2019	10m	The map combines cocoa plantation data with publicly available satellite imagery in a deep learning framework and creates high-resolution maps of cocoa plantations, validated in situ.	https://www.nature.com/articles/s43016-023-00751-8	Support layer
**3**	Global Forest Management Data	2015	100m	The first reference data set and a prototype of a globally consistent forest management map with high spatial detail on the most prevalent forest management classes such as intact forests managed forests with natural regeneration, planted forests, plantation forest (rotation up to 15 years), oil palm plantations, and agroforestry.	https://www.nature.com/articles/s41597-022-01332-3	Support layer
**4**	Forest Landscape Integrity Index	2019	300m	The map integrating data on observed and inferred human pressures and an index of lost connectivity generated a globally consistent, continuous index of forest condition as determined by the degree of anthropogenic modification.	https://www.nature.com/articles/s41467-020-19493-3	Support layer
**5**	Livestock Distribution Map	2015	10km	This dataset contains the reference year 2015 for the following species: cattle, sheep, goats, buffaloes, horses, pigs, chickens, and ducks.	https://dataverse.harvard.edu/dataverse/glw_4	Support layer
**6**	Potential natural vegetations	/	250m	The map used a comparable thematic legend with Copernicus Global Land Operations product to produce high-resolved potential land cover / natural vegetations map.	https://zenodo.org/record/3631254#.ZBxsMuyZPjg	Support layer
**7**	FAOSTAT	2015-2020	Country specific	FAOSTAT provides free access to food and agriculture data for over 245 countries and territories and covers all FAO regional groupings from 1961 to the most recent year available.	https://www.fao.org/faostat/en/	Boundary condition

Table S3. The cause-effect parameters used in modeling MSA.

Drivers	Measurement	Value	MSA	SE
Infrastructure (Primary vegetation)	Distance (m)	<1000	0.4	0.22
	Distance (m)	1000-4000	0.8	0.13
	Distance (m)	4000-14000	0.9	0.06
	Distance (m)	>14000	1	0.02
Infrastructure (Other land use)	Distance (m)	<500	0.4	0.22
	Distance (m)	500-2000	0.8	0.13
	Distance (m)	2000-7000	0.9	0.06
	Distance (m)	>7000	1	0.02
Fragmentation	FFQI	< 0.43	0.3	0.15
	FFQI	0.43 - 0.58	0.6	0.19
	FFQI	0.58 - 0.90	0.7	0.19
	FFQI	0.90 - 0.98	0.9	0.2
	FFQI	0.98 - 0.99	0.95	0.2
	FFQI	> 0.99	1	0.2
Land Use	Water body	0	0	/
	Primary vegetation	1	1	<0.01
	Lightly used natural forest	2	0.7	0.07
	Secondary forest	3	0.5	0.03
	Plantation forest	4	0.2	0.04
	Man-made pastures	6	0.1	0.07
	Agroforestry	7	0.5	0.06
	Low-input agriculture	8	0.3	0.12
	Built-up areas	10	0.05	/

Table S4. The characterization factors (CFs) used for potential disappeared fraction (PDF) calculation.

Ecoregion code	Ecoregion name	Permanent crops taxa-aggregated CFs (PDF/m^2)		
		Median	Lower 95%	Upper 95%
**AT0707**	Guinean forest-savanna mosaic	6.29E-16	-3.34365E-16	2.89861E-15
**AT0111**	Eastern Guinean forests	4.24459E-15	-5.57708E-16	9.05972E-15
**AT0722**	West Sudanian savanna	2.37437E-16	-1.58E-16	1.10474E-15
**AT0130**	Western Guinean lowland forests	3.99521E-15	-1.75132E-15	1.17989E-14
**AT0114**	Guinean montane forests	3.91412E-15	-2.68267E-15	1.35235E-14
**AT1403**	Guinean mangroves	1.71541E-15	-6.62567E-16	5.219E-15

Table S5. Land use allocation results and comparison with other land use datasets.

Land use category	FAOSTAT (kha)	HILDA+ (kha)	GLOBIO4 (kha)	High-resolution land use map (kha)		
**Agriculture**	10030	6737.95	16953.68	11222.61	Agroforestry	3015.27
					Low-input agriculture	8207.34
**Forest**	2949	9262.93	1733.32	3890.32	Primary forest	470.12
					Lightly used natural forest	1134.86
					Secondary forest	2251.13
					Plantation forest	34.21
**Pastures**	13604	13596.46	13410.07	13604.03	Man-made pastures	13604.03
**Urban**	/	272.13	166.53	389.75	Built-up areas	389.75
**Primary vegetation**	/	2596.05	148.91	2431.70	Primary vegetation	2431.70

Table S6. Departmental statistics of cocoa cultivation area in Côte d'Ivoire.

Department*	Full-sun cocoa area [kha]	Agroforestry cocoa area [kha]	Total cocoa area [kha]
SAN-PEDRO	224.45	54.18	278.63
SASSANDRA	215.01	26.94	241.94
TABOU	157.52	51.64	209.16
GAGNOA	168.97	27.90	196.86
GUIGLO	135.59	19.27	154.87
SOUBRE	130.69	22.17	152.86
ABENGOUROU	105.02	47.73	152.75
MEAGUI	134.65	14.69	149.34
VAVOUA	60.88	81.66	142.54
LAKOTA	117.26	24.04	141.30
BLOLEQUIN	88.19	50.74	138.92
DUEKOUE	70.67	59.59	130.25
AGBOVILLE	94.12	21.10	115.22
DALOA	60.79	48.24	109.03
ISSIA	57.43	50.42	107.85
DIVO	98.92	4.28	103.20
BANGOLO	46.93	54.46	101.40
DANANE	38.67	57.85	96.51
ABOISSO	35.48	58.65	94.13
GUITRY	90.37	3.67	94.05
OUME	67.92	6.84	74.75
TIASSALE	72.43	2.32	74.75
MAN	19.79	52.06	71.84
KOUIBLY	21.13	47.67	68.80
AKOUPE	56.08	12.11	68.19
BUYO	34.31	33.29	67.60
ZOUKOUGBEU	28.33	38.95	67.27
YAKASSE-ATTOBROU	30.43	27.28	57.70
BOUAFLE	49.51	6.98	56.49
FRESCO	54.66	1.77	56.43
GRAND-LAHOU	51.78	3.88	55.65
BIANKOUMA	8.81	35.13	43.94
ADZOPE	20.07	22.21	42.28
BETTIE	18.51	23.33	41.84
ZOUAN-HOUNIEN	13.24	27.71	40.95
AGNIBILEKRO	38.05	2.26	40.31
TAI	21.48	16.82	38.29
GUEYO	35.63	2.09	37.71
SINFRA	33.90	3.46	37.36

ALEPE	31.37	4.61	35.98
M'BATTO	25.06	10.22	35.27
ARRAH	15.90	19.18	35.08
FACOBLY	14.18	18.26	32.44
BONGOUANOU	18.37	10.67	29.04
SEGUELA	15.20	9.56	24.77
ZUENOULA	15.90	7.13	23.02
DAOUKRO	12.99	6.98	19.97
KOUN-FAO	16.39	1.91	18.30
SIPILOU	7.98	8.82	16.80
TAABO	14.44	1.38	15.83
TOULEUPLEU	5.17	10.55	15.71
SIKENSI	14.78	0.27	15.05
TOUMODI	4.86	9.85	14.71
TRANSUA	12.90	0.85	13.76
TIAPOUM	6.61	5.61	12.23
YAMOUSSOUKRO	3.24	8.91	12.15
DJEKANOU	0.38	8.81	9.20
BOCANDA	4.17	1.14	5.32
TOUBA	3.23	0.39	3.62
DABOU	1.73	0.58	2.31
KANI	1.48	0.66	2.14
ABIDJAN	1.03	0.47	1.50
PRIKRO	1.01	0.48	1.48

* Only includes departments with an area greater than 1Kha.

Table S7. Departmental statistics of cocoa production in Côte d'Ivoire.

Department*	Agroforestry cocoa production [ton]	Full-sun cocoa production [ton]	Total cocoa production [ton]
SAN-PEDRO	25609.92	110486.63	136096.55
SASSANDRA	13075.19	111252.98	124328.17
GAGNOA	12762.56	78274.95	91037.50
TABOU	22271.44	63563.28	85834.72
ABENGOUROU	26192.27	56618.56	82810.83
GUIGLO	9824.27	71986.69	81810.96
MEAGUI	7213.22	66448.31	73661.53
LAKOTA	12787.39	58644.54	71431.93
SOUBRE	10638.11	60582.39	71220.49
DUEKOUE	31671.78	36807.65	68479.42
BLOLEQUIN	23369.65	41720.84	65090.50
AGBOVILLE	11579.18	52540.62	64119.80
VAVOUA	36601.08	26150.10	62751.18
ABOISSO	34617.92	21035.44	55653.36
ISSIA	25827.98	28715.46	54543.43
DALOA	23750.34	30362.71	54113.05
DIVO	2296.73	51516.06	53812.79
GUITRY	2116.17	51243.34	53359.51
BANGOLO	23521.29	20655.05	44176.33
TIASSALE	1327.55	39286.83	40614.38
DANANE	23371.87	15320.87	38692.74
OUME	3466.75	34565.10	38031.85
AKOUPE	6553.59	30271.04	36824.63
BUYO	18119.54	18282.57	36402.11
ZOUKOUGBEU	20015.06	14512.92	34527.98
YAKASSE-ATTOBROU	15896.99	17697.49	33594.48
FRESCO	934.66	30292.65	31227.32
GRAND-LAHOU	1896.36	28984.71	30881.07
BOUAFLE	3338.06	24925.49	28263.55
MAN	19215.67	6984.60	26200.27
KOUIBLY	17496.31	8116.46	25612.76
BETTIE	13913.56	11124.46	25038.02
ADZOPE	12500.63	11195.55	23696.17
TAI	10076.73	12956.33	23033.05
ALEPE	2844.48	19368.11	22212.60
AGNIBILEKRO	1114.85	19912.20	21027.05

GUEYO	1146.39	19614.76	20761.14
SINFRA	1733.35	16980.28	18713.62
M'BATTO	5403.44	13264.20	18667.64
ARRAH	10157.61	8322.41	18480.02
ZOUAN-HOUNIEN	12172.55	5850.50	18023.05
BONGOUANOU	5643.92	9584.43	15228.35
BIANKOUMA	10656.51	2782.94	13439.45
FACOBLY	6523.97	5267.03	11791.01
ZUENOULA	3361.08	7501.07	10862.15
DAOUKRO	3374.56	6137.11	9511.67
SIKENSI	157.89	8745.80	8903.69
KOUN-FAO	823.46	7801.15	8624.61
SEGUELA	3231.31	5201.65	8432.96
TAABO	715.99	7578.16	8294.14
TOUMODI	4888.61	2490.10	7378.71
TOULEUPLEU	4890.32	2411.73	7302.05
TRANSUA	418.48	6412.59	6831.08
TIAPOUM	3075.69	3615.05	6690.74
YAMOUSSOUKRO	4503.21	1601.79	6105.00
SIPILOU	2584.59	2360.02	4944.61
DJEKANOU	4543.61	197.77	4741.39
BOCANDA	574.86	2044.09	2618.95
DABOU	328.28	997.13	1325.41
TOUBA	125.58	1082.27	1207.86

* Only departments with cocoa production exceeding 1000 tons are included.

Table S8. Departmental statistics of biodiversity impacts of cocoa cultivation in Côte d'Ivoire.

Department*	Lower BIM_{cocoa} [ha]	Upper BIM_{cocoa} [ha]	Lower BIM_{cocoa} relative error [%]	Upper BIM_{cocoa} relative error	BIMcocoa [ha]
TABOU	126853.84	468055.28	-53.33	72.21	271796.94
SASSANDRA	98581.27	441733.34	-57.87	88.78	233998.61
SAN-PEDRO	76642.84	426918.06	-63.47	103.47	209814.27
GUIGLO	86154.09	271184.94	-49.39	59.30	170233.34
MEAGUI	43294.98	254050.02	-64.40	108.92	121604.42
BLOLEQUIN	51904.68	222556.28	-56.71	85.62	119899.18
GAGNOA	34202.21	219186.62	-65.75	119.50	99858.09
DANANE	30159.53	193954.89	-65.85	119.62	88312.21
DUEKOUE	34501.32	153639.23	-58.03	86.89	82209.20
SOUBRE	27327.79	179541.84	-66.60	119.46	81811.41
BANGOLO	30063.76	132626.53	-58.36	83.71	72193.42
LAKOTA	23108.42	161737.12	-67.88	124.83	71937.09
AGBOVILLE	22713.35	128109.95	-63.07	108.28	61508.12
ABENGOUROU	21627.33	128695.77	-64.28	112.53	60554.00
GUITRY	16845.29	109964.69	-66.34	119.71	50050.81
MAN	19031.31	95524.83	-60.89	96.31	48659.57
ABOISSO	17750.27	100100.47	-63.30	106.94	48371.10
DIVO	14201.52	117405.14	-70.64	142.74	48365.65
TIASSALE	17095.33	88244.79	-61.00	101.32	43832.32
TAI	15966.00	85624.63	-62.32	102.06	42375.36
ISSIA	12166.50	98731.12	-70.43	139.98	41141.51
BUYO	12688.27	85548.50	-67.18	121.26	38664.46
VAVOUA	11645.03	79759.96	-67.39	123.36	35708.62
DALOA	10675.82	84764.44	-70.02	138.06	35605.98
GRAND-LAHOU	12447.83	70150.96	-63.21	107.31	33838.38
OUME	10174.83	77258.80	-69.45	131.97	33305.64
YAKASSE-ATTOBROU	12031.83	63326.24	-62.05	99.73	31706.52
KOUIBLY	12637.84	59516.97	-59.68	89.90	31340.80
ZOUKOUGBEU	9832.24	65185.38	-67.16	117.70	29942.47
FRESCO	9067.99	63815.46	-67.72	127.16	28092.84
AKOUPE	9423.78	61033.06	-66.02	120.06	27735.18
GUEYO	12010.69	46406.72	-53.49	79.70	25824.39
BETTIE	10435.89	48957.62	-59.31	90.87	25649.10
BIANKOUMA	9437.71	51927.09	-62.56	106.00	25206.95
ZOUAN-HOUNIEN	8457.64	53882.83	-65.57	119.33	24566.46

BOUAFLE	6698.64	49111.47	-68.81	128.67	21477.45
ALEPE	7217.59	44794.95	-65.46	114.35	20898.27
ADZOPE	4484.68	35743.81	-70.16	137.82	15030.00
SINFRA	3949.88	33373.35	-71.46	141.13	13840.39
AGNIBILEKRO	3606.98	28959.24	-70.21	139.15	12109.38
M'BATTO	3690.87	28054.34	-69.20	134.10	11983.72
TOULEUPLEU	4580.53	22450.43	-59.16	100.16	11216.52
FACOBLY	3445.63	21373.19	-65.34	114.99	9941.37
SIKENSI	3587.61	19125.16	-62.08	102.14	9461.28
ARRAH	2792.39	22398.48	-70.38	137.62	9426.08
TIAPOUM	3735.82	14414.49	-54.48	75.65	8206.51
SIPILOU	3045.34	15123.18	-60.41	96.58	7692.98
BONGOUANOU	1517.35	17811.55	-77.31	166.33	6687.72
TAABO	1970.93	15374.66	-69.76	135.91	6517.06
ZUENOULA	1745.39	12560.35	-68.35	127.79	5513.90
TOUMODI	1337.55	9741.79	-68.51	129.32	4248.11
DJEKANOU	1644.64	7215.34	-57.34	87.17	3854.89
DAOUKRO	747.51	9794.22	-79.08	174.09	3573.36
SEGUELA	920.96	7477.73	-70.97	135.69	3172.71
KOUN-FAO	542.68	9126.75	-81.87	204.95	2992.89
YAMOUSSOUKRO	743.00	7423.98	-74.78	151.97	2946.39
TRANSUA	354.09	6443.98	-82.78	213.35	2056.51
DABOU	121.93	2093.50	-84.69	162.92	796.25
BOCANDA	37.58	2395.62	-94.96	221.17	745.90

* Only departments with cocoa production exceeding 1000 tons are included.

Table S9. Departmental statistics of land-use-related biodiversity impacts per ton of cocoa produced in Côte d'Ivoire.

Department*	Lower BF_{cocoa} [ha·year·ton^{-1}]	Upper BF_{cocoa} [ha·year·ton^{-1}]	BF_{cocoa} [ha·year·ton^{-1}]
SAN-PEDRO	0.57	3.15	1.55
SASSANDRA	0.80	3.57	1.89
GAGNOA	0.38	2.42	1.10
TABOU	1.49	5.48	3.18
ABENGOUROU	0.26	1.52	0.71
GUIGLO	1.06	3.34	2.10
MEAGUI	0.59	3.46	1.66
LAKOTA	0.32	2.27	1.01
SOUBRE	0.38	2.53	1.15
DUEKOUE	0.51	2.26	1.21
BLOLEQUIN	0.80	3.45	1.86
AGBOVILLE	0.35	2.00	0.96
VAVOUA	0.19	1.28	0.57
ABOISSO	0.31	1.72	0.83
ISSIA	0.22	1.82	0.76
DALOA	0.20	1.57	0.66
DIVO	0.26	2.18	0.90
GUITRY	0.32	2.06	0.94
BANGOLO	0.69	3.02	1.65
TIASSALE	0.42	2.18	1.08
DANANE	0.79	5.06	2.30
OUME	0.27	2.04	0.88
AKOUPE	0.26	1.66	0.75
BUYO	0.35	2.36	1.07
ZOUKOUGBEU	0.29	1.90	0.87
YAKASSE-ATTOBROU	0.36	1.89	0.94
FRESCO	0.29	2.04	0.90
GRAND-LAHOU	0.40	2.27	1.10
BOUAFLE	0.24	1.74	0.76
MAN	0.73	3.67	1.87
KOUIBLY	0.50	2.33	1.23
BETTIE	0.42	1.95	1.02
ADZOPE	0.19	1.51	0.63
TAI	0.70	3.73	1.85
AGNIBILEKRO	0.16	1.30	0.54

ALEPE	0.32	2.02	0.94
GUEYO	0.58	2.24	1.25
SINFRA	0.21	1.79	0.74
M'BATTO	0.20	1.50	0.64
ARRAH	0.15	1.21	0.51
ZOUAN-HOUNIEN	0.47	3.01	1.37
BONGOUANOU	0.10	1.17	0.44
BIANKOUMA	0.71	3.90	1.89
FACOBLY	0.29	1.83	0.85
ZUENOULA	0.16	1.16	0.51
DAOUKRO	0.08	1.03	0.38
SIKENSI	0.40	2.15	1.06
KOUN-FAO	0.06	1.04	0.34
SEGUELA	0.11	0.89	0.38
TAABO	0.24	1.86	0.79
TIAPOUM	0.48	1.85	1.05
TRANSUA	0.05	0.86	0.28
TOUMODI	0.18	1.32	0.58
TOULEUPLEU	0.63	3.11	1.55
YAMOUSSOUKRO	0.12	1.22	0.48
SIPILOU	0.62	3.07	1.56
DJEKANOU	0.35	1.52	0.81
BOCANDA	0.01	0.92	0.29
DABOU	0.09	1.58	0.60
TOUBA	0.21	1.02	0.52

* Only departments with cocoa production exceeding 1000 tons are included.

Table S10. Statistics of land-use-related biodiversity impacts and biodiversity impacts per ton of cocoa imported for importing countries.

Country or region	$BF_{cocoa, import}$ [ha·year·ton^{-1}]	Spatially explicit $BF_{cocoa, import}$ [ha·year·ton^{-1}]	BIM [ha·year]	PDF $BF_{cocoa, import}$ [10^{-11} PDF·year·ton^{-1}]	Spatially explicit PDF $BF_{cocoa, import}$ [10^{-11} PDF·year·ton^{-1}]	PDF [10^{-11} PDF·year]
Netherlands	1.24	1.19	676189.93	8.21	8.16	4466134.72
United States	1.22	1.16	284366.64	8.17	8.08	1893270.71
Belgium	1.23	1.18	221204.37	8.21	8.16	1467719.90
Malaysia	1.29	1.31	201641.95	8.23	8.20	1282093.89
Germany	1.22	1.11	183735.63	8.19	8.06	1226042.60
France	1.20	1.16	141343.81	8.16	8.11	957831.61
United Kingdom	1.25	1.21	114628.75	8.23	8.21	754430.80
Estonia	1.27	1.26	100344.87	8.26	8.26	648980.13
Indonesia	1.37	1.49	92492.13	8.34	8.47	563014.45
Canada	1.28	1.27	83014.89	8.25	8.26	534098.55
Turkey	1.26	1.19	80963.87	8.25	8.26	525778.67
Italy	1.24	1.20	66992.79	8.21	8.16	440785.14
Brazil	1.24	1.19	51768.80	8.22	8.18	342717.08
Spain	1.31	1.37	52995.55	8.20	8.09	330781.67
Poland	1.18	1.15	24227.81	8.07	8.03	165748.52
Mexico	1.25	1.24	24542.24	8.27	8.28	161553.75
Bulgaria	1.31	1.38	23260.06	8.29	8.38	146960.54
China	1.29	1.39	21490.85	8.27	8.41	136596.94
Ghana	1.26	1.24	15168.02	8.23	8.20	98487.48
United Arab Emirates	1.26	1.24	8846.60	8.22	8.19	57498.51
India	1.28	1.26	6453.55	8.26	8.28	41509.12
Israel	1.20	1.17	5779.82	8.21	8.19	39359.79
Tunisia	1.29	1.33	4830.98	8.27	8.33	30863.50
Australia	1.21	1.18	3285.91	8.21	8.20	22254.18
Singapore	1.27	1.26	2924.73	8.26	8.27	19036.16
Portugal	1.27	1.26	1864.53	8.26	8.27	12087.78
Japan	1.27	1.26	1371.26	8.26	8.27	8937.08
South Africa	1.25	1.23	1234.39	8.25	8.24	8148.67
Senegal	1.19	1.14	845.53	8.20	8.16	5794.40
Chile	1.26	1.26	618.10	8.26	8.26	4036.70
Russia	1.27	1.26	447.15	8.26	8.27	2910.37
Syria	1.27	1.26	383.27	8.26	8.27	2494.60
Sri Lanka	1.28	/	256.06	8.25	/	1641.64
Algeria	1.24	1.14	162.80	8.23	8.16	1071.34

Taiwan	1.18	1.14	89.43	8.19	8.16	617.95
Greece	1.27	1.26	86.69	8.26	8.27	564.25
Togo	1.28	/	69.09	8.25	/	442.97
Morocco	1.28	/	61.39	8.25	/	393.60
Lithuania	1.28	/	30.70	8.25	/	196.80
Saudi Arabia	1.27	1.26	27.38	8.26	8.27	178.19
Lebanon	1.28	/	19.96	8.25	/	127.96
Egypt	1.27	1.26	4.56	8.26	8.27	29.70

Table S11. Departmental statistics of biodiversity impact indicators in Côte d'Ivoire. The biodiversity impact indicators included as follows: production weighted average MSA loss, production-weighted average RI, and BF_{cocoa}.

Department*	Agroforestry MSA loss	Full-sun MSA loss	Cocoa MSA loss	Agroforestry RI	Full-sun RI	Cocoa RI	Agroforestry BF_{cocoa}	Full-sun BF_{cocoa}	BF_{cocoa}
SAN-PEDRO	0.69	0.89	0.85	3.81	3.60	3.64	1.32	1.59	1.32
SASSANDRA	0.74	0.98	0.95	7.84	3.63	4.07	3.11	1.74	3.11
GAGNOA	0.71	0.85	0.83	2.89	2.84	2.85	0.93	1.12	0.93
TABOU	0.96	1.34	1.24	7.06	5.97	6.26	2.98	3.23	2.98
ABENGOUROU	0.69	0.69	0.69	2.04	1.90	1.95	0.78	0.71	0.78
GUIGLO	0.97	1.08	1.07	3.53	3.68	3.66	1.76	2.12	1.76
MEAGUI	0.80	0.84	0.84	4.27	3.81	3.86	1.77	1.64	1.77
LAKOTA	0.65	0.78	0.75	2.50	2.68	2.64	0.87	1.04	0.87
SOUBRE	0.57	0.88	0.83	2.78	2.99	2.96	0.77	1.21	0.77
DUEKOUE	0.68	0.91	0.80	2.75	2.86	2.81	1.01	1.37	1.01
BLOLEQUIN	0.85	1.00	0.94	4.23	4.14	4.17	1.66	1.95	1.66
AGBOVILLE	0.61	0.72	0.70	2.42	2.48	2.47	0.80	0.99	0.80
VAVOUA	0.58	0.75	0.65	1.97	1.87	1.93	0.53	0.62	0.53
ABOISSO	0.55	0.62	0.57	2.58	2.59	2.58	0.82	0.95	0.82
ISSIA	0.44	0.68	0.57	2.55	2.66	2.61	0.58	0.91	0.58
DALOA	0.50	0.67	0.60	2.22	2.21	2.21	0.55	0.74	0.55
DIVO	0.51	0.64	0.63	2.75	2.73	2.73	0.74	0.91	0.74
GUITRY	0.54	0.68	0.68	2.40	2.45	2.45	0.75	0.95	0.75
BANGOLO	0.91	1.10	1.00	3.68	3.72	3.70	1.47	1.82	1.47
TIASSALE	0.81	0.78	0.78	2.56	2.51	2.52	1.20	1.08	1.20
DANANE	0.77	0.95	0.84	7.72	7.25	7.53	2.17	2.46	2.17
OUME	0.58	0.70	0.69	2.45	2.48	2.48	0.72	0.89	0.72
AKOUPE	0.58	0.71	0.69	2.09	2.02	2.03	0.65	0.78	0.65
BUYO	0.57	0.70	0.63	2.96	3.17	3.06	0.94	1.19	0.94
ZOUKOUGBEU	0.64	0.70	0.66	2.54	2.54	2.54	0.84	0.91	0.84
YAKASSE-ATTOBROU	0.64	0.70	0.67	2.43	2.42	2.43	0.91	0.98	0.91
FRESCO	0.47	0.67	0.66	2.52	2.45	2.46	0.63	0.91	0.63
GRAND-LAHOU	0.71	0.75	0.75	3.01	2.66	2.68	1.07	1.10	1.07
BOUAFLE	0.52	0.68	0.66	2.15	2.28	2.26	0.55	0.79	0.55
MAN	0.87	1.30	0.98	4.76	5.84	5.05	1.55	2.71	1.55
KOUIBLY	1.02	1.26	1.10	3.20	2.69	3.04	1.20	1.28	1.20
BETTIE	0.63	0.74	0.68	2.45	2.58	2.50	0.92	1.15	0.92
ADZOPE	0.43	0.58	0.50	2.38	2.18	2.29	0.57	0.71	0.57
TAI	0.65	0.59	0.62	5.07	4.77	4.90	2.01	1.71	2.01
ALEPE	0.35	0.61	0.58	2.65	2.63	2.63	0.57	0.99	0.57

AGNIBILEKRO	0.44	0.64	0.63	1.69	1.72	1.72	0.37	0.59	0.37
GUEYO	0.72	0.92	0.91	2.46	2.48	2.48	0.98	1.26	0.98
SINFRA	0.48	0.65	0.63	2.35	2.34	2.34	0.57	0.76	0.57
M'BATTO	0.50	0.60	0.58	2.07	2.10	2.09	0.56	0.68	0.56
ARRAH	0.51	0.53	0.52	1.88	1.83	1.85	0.51	0.51	0.51
ZOUAN-HOUNIEN	0.67	0.75	0.70	4.45	4.42	4.44	1.31	1.46	1.31
BONGOUANOU	0.38	0.51	0.46	1.87	1.81	1.84	0.37	0.48	0.37
BIANKOUMA	0.97	1.38	1.05	5.51	6.45	5.70	1.64	2.77	1.64
FACOBLY	0.76	1.14	0.93	2.64	2.40	2.53	0.71	1.00	0.71
ZUENOULA	0.56	0.71	0.66	1.59	1.60	1.60	0.42	0.54	0.42
DAOUKRO	0.39	0.51	0.47	1.72	1.68	1.69	0.32	0.41	0.32
SIKENSI	0.60	0.70	0.69	2.64	2.57	2.58	0.92	1.07	0.92
KOUN-FAO	0.41	0.45	0.45	1.74	1.62	1.63	0.31	0.35	0.31
SEGUELA	0.58	0.97	0.83	1.40	1.36	1.37	0.27	0.44	0.27
TAABO	0.66	0.63	0.64	2.34	2.36	2.36	0.79	0.78	0.79
TOUMODI	0.48	0.60	0.52	2.26	2.13	2.21	0.54	0.65	0.54
TOULEUPLEU	0.67	0.90	0.74	4.42	4.39	4.41	1.38	1.86	1.38
TRANSUA	0.24	0.44	0.42	1.48	1.43	1.43	0.17	0.31	0.17
TIAPOUM	0.78	0.89	0.84	2.69	2.76	2.72	1.15	1.29	1.15
YAMOUSSOUKRO	0.35	0.74	0.45	2.11	2.13	2.11	0.38	0.78	0.38
SIPILOU	1.17	1.42	1.29	4.10	3.91	4.01	1.43	1.69	1.43
DJEKANOU	0.68	0.57	0.68	2.32	2.26	2.32	0.82	0.67	0.82
BOCANDA	0.24	0.36	0.34	1.71	1.72	1.72	0.20	0.31	0.20
DABOU	0.30	0.41	0.38	2.68	2.74	2.72	0.46	0.65	0.46
TOUBA	0.59	1.28	1.21	1.34	1.30	1.30	0.25	0.55	0.25
National average	0.67	0.82	0.78	3.35	3.05	3.14	1.13	1.30	1.25

* Only departments with cocoa production exceeding 1000 tons are included.

Table S12. Statistics on the biodiversity impacts of Dutch cocoa imports from Côte d'Ivoire for each cocoa production departments in absolute and relative perspectives (only includes the share that can be traced back to specific production departments).

Department	Absolute biodiversity impacts [ha·year]	Relative biodiversity impacts [%]
TABOU	31434.79	2.76
SAN-PEDRO	30436.34	2.80
DUEKOUE	23830.20	6.04
SASSANDRA	23420.57	2.80
MEAGUI	16724.51	3.31
DIVO	13113.74	2.85
GAGNOA	10915.31	1.94
LAKOTA	10323.93	3.06
SOUBRE	10158.77	2.34
DALOA	10146.59	2.61
BLOLEQUIN	8782.38	1.70
BIANKOUMA	8142.26	2.11
YAKASSE-ATTOBROU	8111.18	3.95
ABOISSO	8011.23	1.21
ABENGOUROU	7439.66	1.90
MAN	6326.70	1.57
BANGOLO	5938.89	2.15
BOUAFLE	5513.65	1.40
GUITRY	5272.28	1.48
OUME	5238.63	1.90
FRESCO	4648.73	2.00
GUIGLO	3907.28	0.69
ALEPE	3857.25	0.89
VAVOUA	3229.58	0.70
DANANE	3197.44	0.29
ADZOPE	2904.54	1.14
AGBOVILLE	2884.49	0.54
SINFRA	2533.40	1.43
TAI	2081.41	0.27
ISSIA	1972.36	0.43
BETTIE	1904.09	1.34
TIASSALE	1691.23	0.57
GRAND-LAHOU	1635.89	0.21

GUEYO	1554.29	1.76
BUYO	1526.32	0.39
DJEKANOU	1512.38	3.65
SIPILOU	1387.84	1.27
AGNIBILEKRO	1360.65	0.94
ZOUAN-HOUNIEN	1158.27	0.50
SIKENSI	1151.51	1.03
ARRAH	1140.65	0.81
YAMOUSSOUKRO	954.93	0.67
AKOUPE	736.36	0.43
TAABO	730.70	0.57
FACOBLY	657.76	0.75
TIAPOUM	614.97	0.17
KOUIBLY	600.86	0.46
ZOUKOUGBEU	521.44	0.27
TOUMODI	390.83	0.16
ABIDJAN	366.21	0.05
M'BATTO	347.47	0.16
ZUENOULA	278.03	0.15
KANI	218.05	0.16
BONGOUANOU	191.36	0.14
SEGUELA	164.59	0.06

Table S13. The upper and lower bounds of the biodiversity impacts of cocoa imports in different importing countries.

Country or region	Lower $BF_{cocoa, import}$ [ha·year·ton^{-1}]	Lower spatially explicit $BF_{cocoa, import}$ [ha·year·ton^{-1}]	Upper $BF_{cocoa, import}$ [ha·year·ton^{-1}]	Upper spatially explicit $BF_{cocoa, import}$ [ha·year·ton^{-1}]	Lower BIM_{cocoa} [ha·year]	Upper BIM_{cocoa} [ha·year]
Netherlands	0.47	0.45	2.51	2.45	258588.39	1370181.92
United States	0.47	0.44	2.48	2.39	108616.83	576743.92
Belgium	0.47	0.44	2.50	2.42	84656.23	447857.85
Malaysia	0.5	0.51	2.59	2.64	78151.97	404863.55
Germany	0.47	0.42	2.46	2.27	70803.85	370646.60
France	0.45	0.43	2.46	2.40	53253.61	289189.60
United Kingdom	0.48	0.46	2.52	2.48	43881.89	232051.78
Estonia	0.49	0.48	2.56	2.54	38778.37	201952.14
Indonesia	0.53	0.58	2.73	2.95	36092.17	184715.63
Canada	0.49	0.49	2.57	2.57	32159.51	166739.92
Turkey	0.49	0.46	2.54	2.41	31398.62	162641.20
Italy	0.48	0.46	2.50	2.42	25874.35	134758.34
Spain	0.47	0.45	2.51	2.46	19740.47	105086.28
Brazil	0.51	0.55	2.60	2.67	20855.32	105318.99
Mexico	0.44	0.42	2.43	2.40	9003.81	49942.93
Poland	0.48	0.46	2.56	2.55	9317.11	50028.08
Bulgaria	0.51	0.55	2.60	2.71	9105.1	46387.97
China	0.5	0.54	2.59	2.76	8359.06	43054.92
Ghana	0.49	0.47	2.54	2.50	5862.31	30514.29
United Arab Emirates	0.48	0.47	2.55	2.52	3397.37	17864.79
India	0.49	0.47	2.57	2.61	2490.3	13003.17
Israel	0.45	0.43	2.46	2.42	2175.39	11836.63
Tunisia	0.5	0.52	2.58	2.63	1880.47	9673.79
Australia	0.46	0.44	2.48	2.44	1238.84	6720.62
Singapore	0.48	0.48	2.57	2.57	1115.88	5927.21
Portugal	0.49	0.48	2.57	2.57	714.84	3768.64
Japan	0.48	0.48	2.57	2.57	522.98	2779.83
South Africa	0.47	0.46	2.54	2.52	469	2509.71
Senegal	0.45	0.42	2.45	2.37	318.13	1732.88
Chile	0.48	0.48	2.56	2.56	235.59	1253.61
Russia	0.48	0.48	2.57	2.57	170.6	906.19
Syria	0.48	0.48	2.57	2.57	146.23	776.73
Sri Lanka	0.5	/	2.57	/	99.5	513.71
Algeria	0.48	0.42	2.52	2.37	62.49	329.32
Taiwan	0.44	0.42	2.43	2.37	33.51	183.79
Greece	0.48	0.48	2.57	2.57	33.08	175.69

Togo	0.5	/	2.57	/	26.85	138.62
Morocco	0.5	/	2.57	/	23.86	123.17
Lithuania	0.5	/	2.57	/	11.93	61.58
Saudi Arabia	0.48	0.48	2.57	2.57	10.45	55.48
Lebanon	0.5	/	2.57	/	7.76	40.04
Egypt	0.48	0.48	2.57	2.57	1.74	9.25

REFERENCES

(1) *GADM*. https://gadm.org/index.html (accessed 2023-03-20).

(2) Schipper, A. M.; Hilbers, J. P.; Meijer, J. R.; Antão, L. H.; Benítez-López, A.; de Jonge, M. M. J.; Leemans, L. H.; Scheper, E.; Alkemade, R.; Doelman, J. C.; Mylius, S.; Stehfest, E.; van Vuuren, D. P.; van Zeist, W.-J.; Huijbregts, M. A. J. Projecting Terrestrial Biodiversity Intactness with GLOBIO 4. *Glob. Change Biol.* **2020**, *26* (2), 760–771. https://doi.org/10.1111/gcb.14848.

(3) Parra-Paitan, C.; Verburg, P. H. Accounting for Land Use Changes beyond the Farm-Level in Sustainability Assessments: The Impact of Cocoa Production. *Sci. Total Environ.* **2022**, *825*, 154032. https://doi.org/10.1016/j.scitotenv.2022.154032.

(4) *GLW 4: Gridded Livestock Density (Global - 2015 - 10 km) - "FAO catalog."* https://data.apps.fao.org/catalog/iso/15f8c56c-5499-45d5-bd89-59ef6c026704 (accessed 2023-03-19).

(5) Robinson, T. P.; Thorton, P.; Franceschini, G.; Kruska, R.; Chiozza, F.; Notenbaert, A.; Cecchi, G.; Herrero, M.; Epprecht, M.; Fritz, S.; You, L.; Conchedda, G.; See, L. *Global Livestock Production Systems*; FAO/ILRI, 2011. http://www.fao.org/docrep/014/i2414e/i2414e00.htm (accessed 2023-03-20).

(6) Lesiv, M.; Schepaschenko, D.; Buchhorn, M.; See, L.; Dürauer, M.; Georgieva, I.; Jung, M.; Hofhansl, F.; Schulze, K.; Bilous, A.; Blyshchyk, V.; Mukhortova, L.; Brenes, C. L. M.; Krivobokov, L.; Ntie, S.; Tsogt, K.; Pietsch, S. A.; Tikhonova, E.; Kim, M.; Di Fulvio, F.; Su, Y.-F.; Zadorozhniuk, R.; Sirbu, F. S.; Panging, K.; Bilous, S.; Kovalevskii, S. B.; Kraxner, F.; Rabia, A. H.; Vasylyshyn, R.; Ahmed, R.; Diachuk, P.; Kovalevskyi, S. S.; Bungnamei, K.; Bordoloi, K.; Churilov, A.; Vasylyshyn, O.; Sahariah, D.; Tertyshnyi, A. P.; Saikia, A.; Malek, Ž.; Singha, K.; Feshchenko, R.; Prestele, R.; Akhtar, I. ul H.; Sharma, K.; Domashovets, G.; Spawn-Lee, S. A.; Blyshchyk, O.; Slyva, O.; Ilkiv, M.; Melnyk, O.; Sliusarchuk, V.; Karpuk, A.; Terentiev, A.; Bilous, V.; Blyshchyk, K.; Bilous, M.; Bogovyk, N.; Blyshchyk, I.; Bartalev, S.; Yatskov, M.; Smets, B.; Visconti, P.; Mccallum, I.; Obersteiner, M.; Fritz, S. Global Forest Management Data for 2015 at a 100 m Resolution. *Sci. Data* **2022**, *9* (1), 199. https://doi.org/10.1038/s41597-022-01332-3.

(7) Grantham, H. S.; Duncan, A.; Evans, T. D.; Jones, K. R.; Beyer, H. L.; Schuster, R.; Walston, J.; Ray, J. C.; Robinson, J. G.; Callow, M.; Clements, T.; Costa, H. M.; DeGemmis, A.; Elsen, P. R.; Ervin, J.; Franco, P.; Goldman, E.; Goetz, S.; Hansen, A.; Hofsvang, E.; Jantz, P.; Jupiter, S.; Kang, A.; Langhammer, P.; Laurance, W. F.; Lieberman, S.; Linkie, M.; Malhi, Y.; Maxwell, S.; Mendez, M.; Mittermeier, R.; Murray, N. J.; Possingham, H.; Radachowsky, J.; Saatchi, S.; Samper, C.; Silverman, J.; Shapiro, A.; Strassburg, B.; Stevens, T.; Stokes, E.; Taylor, R.; Tear, T.; Tizard, R.; Venter, O.; Visconti, P.; Wang, S.; Watson, J. E. M. Anthropogenic Modification of Forests Means Only 40% of Remaining Forests Have High Ecosystem Integrity. *Nat. Commun.* **2020**, *11* (1), 5978. https://doi.org/10.1038/s41467-020-19493-3.

(8) Alkemade, R.; van Oorschot, M.; Miles, L.; Nellemann, C.; Bakkenes, M.; ten Brink, B. GLOBIO3: A Framework to Investigate Options for Reducing Global Terrestrial Biodiversity Loss. *Ecosystems* **2009**, *12* (3), 374–390. https://doi.org/10.1007/s10021-009-9229-5.

(9) Zaks, I. *InVEST*. Natural Capital Project. https://naturalcapitalproject.stanford.edu/software/invest (accessed 2023-03-21).

(10) Chaudhary, A.; Verones, F.; de Baan, L.; Hellweg, S. Quantifying Land Use Impacts on Biodiversity: Combining Species–Area Models and Vulnerability Indicators. *Environ. Sci. Technol.* **2015**, *49* (16), 9987–9995. https://doi.org/10.1021/acs.est.5b02507.

(11) *Inventaire forestier et faunique national (IFFN) de la Côte d'Ivoire*. ONF International. https://www.onfinternational.org/projets/iffn-rci/ (accessed 2024-01-10).

(12) Winkler, K.; Fuchs, R.; Rounsevell, M.; Herold, M. Global Land Use Changes Are Four Times Greater than Previously Estimated. *Nat. Commun.* **2021**, *12* (1), 2501. https://doi.org/10.1038/s41467-021-22702-2.
(13) Läderach, P.; Martinez-Valle, A.; Schroth, G.; Castro, N. Predicting the Future Climatic Suitability for Cocoa Farming of the World's Leading Producer Countries, Ghana and Côte d'Ivoire. *Clim. Change* **2013**, *119* (3), 841–854. https://doi.org/10.1007/s10584-013-0774-8.
(14) Institute, I. F. P. R. Spatially-Disaggregated Crop Production Statistics Data in Africa South of the Sahara for 2017, 2020. https://doi.org/10.7910/DVN/FSSKBW.
(15) Abu, I.-O.; Szantoi, Z.; Brink, A.; Robuchon, M.; Thiel, M. Detecting Cocoa Plantations in Côte d'Ivoire and Ghana and Their Implications on Protected Areas. *Ecol. Indic.* **2021**, *129*, 107863. https://doi.org/10.1016/j.ecolind.2021.107863.
(16) Kalischek, N.; Lang, N.; Renier, C.; Daudt, R. C.; Addoah, T.; Thompson, W.; Blaser-Hart, W. J.; Garrett, R.; Schindler, K.; Wegner, J. D. Cocoa Plantations Are Associated with Deforestation in Côte d'Ivoire and Ghana. *Nat. Food* **2023**, *4* (5), 384–393. https://doi.org/10.1038/s43016-023-00751-8.
